# From Therapeutic Factors to Mechanisms of Change in the Creative Arts Therapies: A Scoping Review

**DOI:** 10.3389/fpsyg.2021.678397

**Published:** 2021-07-15

**Authors:** Martina de Witte, Hod Orkibi, Rebecca Zarate, Vicky Karkou, Nisha Sajnani, Bani Malhotra, Rainbow Tin Hung Ho, Girija Kaimal, Felicity A. Baker, Sabine C. Koch

**Affiliations:** ^1^Research Institute of Child Development and Education, University of Amsterdam, Amsterdam, Netherlands; ^2^Kennisontwikkeling Vaktherapieën (KenVaK) Research Centre for the Arts Therapies, Heerlen, Netherlands; ^3^Hogeschool van Arnhem en Nijmegen University of Applied Sciences, Nijmegen, Netherlands; ^4^Stevig Specialized and Forensic Care for Patients With Intellectual Disabilities, Dichterbij, Oostrum, Netherlands; ^5^Faculty of Social Welfare and Health Sciences, Emili Sagol Creative Arts Therapies Research Center, University of Haifa, Haifa, Israel; ^6^Division of Expressive Therapies, Lesley University, Cambridge, MA, United States; ^7^Research Centre for Arts and Wellbeing, Edge Hill University, Ormskirk, United Kingdom; ^8^Educational Theatre & Rehabilitation Science Ph.D. Programs, New York University, New York, NY, United States; ^9^Department of Creative Arts Therapies, Drexel University, Philadelphia, PA, United States; ^10^Department of Social Work and Social Administration, Centre on Behavioral Health, The University of Hong Kong, Hong Kong, China; ^11^Music Therapy Department, Norwegian Academy of Music, Oslo, Norway; ^12^Faculty of Fine Arts and Music, The University of Melbourne, Parkville, VIC, Australia; ^13^SRH University Heidelberg, Heidelberg, Germany; ^14^Department for Creative Arts Therapies and Therapy Science, Alanus University of Arts and Social Sciences, Alfter, Germany

**Keywords:** creative arts therapies, mechanisms of change, therapeutic factors, drama therapy, dance movement therapy, psychodrama, music therapy, art therapy

## Abstract

Empirical studies in the creative arts therapies (CATs; i.e., art therapy, dance/movement therapy, drama therapy, music therapy, psychodrama, and poetry/bibliotherapy) have grown rapidly in the last 10 years, documenting their positive impact on a wide range of psychological and physiological outcomes (e.g., stress, trauma, depression, anxiety, and pain). However, it remains unclear *how* and *why* the CATs have positive effects, and which therapeutic factors account for these changes. Research that specifically focuses on the therapeutic factors and/or mechanisms of change in CATs is only beginning to emerge. To gain more insight into how and why the CATs influence outcomes, we conducted a scoping review (*N*_studies_ = 67) to pinpoint therapeutic factors specific to each CATs discipline, joint factors of CATs, and more generic common factors across all psychotherapy approaches. This review therefore provides an overview of empirical CATs studies dealing with therapeutic factors and/or mechanisms of change, and a detailed analysis of these therapeutic factors which are grouped into domains. A framework of 19 domains of CATs therapeutic factors is proposed, of which the three domains are composed solely of factors unique to the CATs: “embodiment,” “concretization,” and “symbolism and metaphors.” The terminology used in change process research is clarified, and the implications for future research, clinical practice, and CATs education are discussed.

## Introduction

There is growing recognition of the role played by different art forms in improving health and well-being (Clift and Camic, 2016; Fancourt and Finn, 2019; Nitzan and Orkibi, 2020; Sonke et al., 2021), whether as part of everyday life (not for health purposes but with a secondary health benefit), within arts programs designed to promote health, or within specific therapeutic interventions provided by qualified creative arts therapists in various kinds of healthcare settings. The creative arts therapies (CATs) are characterized by the clinical and evidence-informed use of the arts within a therapeutic relationship that relies on experiential and action-based interventions (De Witte et al., 2020c). In recent years it has also become apparent that, for example, in addition to cognitive-behavioral therapy (CBT), there is a clear need for more empirical evidence on the effects and applicability of therapeutic interventions rooted in the action-based approach such as CATs. Some studies on CBT have shown that behavioral activation strategies alone, such as role-play exercises, result in the same outcome as the full cognitive therapy package, especially in client populations with cognitive limitations (Cuijpers et al., 2007; Didden, 2007). This is in line with reviews from psychotherapy research, phenomenology, and cognitive science that have also reported the benefits of action- and experience-based interventions (e.g., Elliott et al., 2013; Fuchs and Koch, 2014; Koch, 2017).

Although the growing number of systematic reviews and meta-analyses of the CATs reflect the increase in individual studies examining the effects of CATs on psychological and physiological outcomes (e.g., Koch et al., 2019; De Witte et al., 2020b), little is known about which change factors of the CATs lead to or impact certain health outcomes. To better understand the CATs-related factors leading to therapeutic change in both physiological and psychological outcomes, we conducted a scoping review of published empirical studies to contribute to disseminating the existing evidence and guide future research.

### The Creative Arts Therapies

The creative arts therapies (CATs) is an umbrella term covering healthcare professions in several disciplines: art therapy, dance/movement therapy, drama therapy, psychodrama, music therapy, and poetry/bibliotherapy (see Table 1). Creative arts therapists complete extensive education and clinical training in using arts-based methods and the creative processes and their outcomes to ameliorate disabilities and illnesses and optimize health and well-being within a therapeutic relationship (https://www.nccata.org/). Creative arts therapists work with clients of all ages, with individuals, dyads, families, and groups across a variety of mental healthcare, medical, rehabilitation, educational, and community settings (Orkibi, 2020). CATs scholars are currently developing the terminology and knowledge base for the unique contribution of CATs as specific fields of psychotherapy (e.g., Baker et al., 2015; Koch, 2017; Dunphy et al., 2019). Within this attempt, there is a growing need to pinpoint not only the effectiveness of the CATs, but also the in-session change processes that lead to desirable health outcomes.

**Table 1:** Description of each CAT discipline.

**CATs discipline**	**Description**
Art therapy (AT)	Uses a spectrum of 2- and 3- dimensional structured and unstructured visual art media (e.g., pencils, paints, chalk, crayons, found objects, clay, fabrics, etc.), within a psychotherapeutic relationship with an art therapist. The art therapist facilitates non-verbal and verbal self-expression and reflection through the process of art making and the resulting artwork.
Dance movement therapy (DMT)	Employs dance and movement as a way into and a means of therapy, within a psychotherapeutic relationship, with the goal of promoting physical, emotional, cognitive, social, and spiritual integration of individuals. It is based on the premise of the interconnection of body and mind.
Drama therapy (DT) and psychodrama (PD)	DT involves the intentional use of drama and theater processes such as embodiment, dramatic projection, improvisation, role-play, and performance to facilitate physiological, psychological, and social change. PD uses guided role-play and specific techniques to explore clients' personal and interpersonal problems and possible solutions. While both operate in a dramatic reality, in DT the story and characters are mostly imaginary, symbolic, and fantasy-based, whereas in PD they are mostly reality-based.
Music therapy (MT)	Uses music and its properties (e.g., melody, rhythm, tempo, dynamics, pitch), as well as song writing, improvisation, and singing within a therapeutic relationship to optimize clients' quality of life and improve their physical, social, communicative, emotional, intellectual, and spiritual health and well-being. MT can involve active music making and/or receptive music listening, according to the client's needs.
Poetry/biblio therapy (P/BT)	Uses written language, poetry writing and reading, expressive writing, journal writing, as well as story writing and reading within a therapeutic relationship.

*From Orkibi (2020)*.

### Theoretical Assumptions and Models of Change of the CATs

To provide a theoretical background on the ways in which CATs interventions may lead to therapeutic change and positive outcomes, the leading theoretical CAT models are briefly described below. Over the last few decades, several theoretical models of change that apply to all CATs specializations have been proposed.

In 1980, Rudolf Arnheim asked: “What is it that endows the arts with the healing powers you see at work?” He specified factors such as hedonism, pleasure, symbolic communication, trying out, and integrity (Arnheim, 1980). A decade later, Blatner (1992) described the underlying therapeutic principles of all CATs as a theory of praxis, and argued that clients project their emotions and ideas into the artistic medium that function as a transitional object. Blatner (1992) also asserted that symbolization, creativity, spontaneity, playfulness, role expansion, and imagery are unique elements of CATs. Johnson (1998) introduced a psychodynamic model involving a sequential process of externalization, transformation, and re-integration to account for therapeutic action in the CATs. Karkou and Sanderson (2006) claimed that the arts in CATs are perceived as a participatory activity without the requirement for artistic quality or the need for a final artistic product; they viewed creativity, imagery, symbolism, metaphor, and non-verbal communication as important agents of therapeutic change across the CATs. Koch (2017) proposed a theoretical model of change emphasizing embodied aesthetics in the CATs in which active art-making (moving) and art-reception (being moved), occur in a cyclic process of expression and impression. Five clusters of CATs therapeutic factors were hypothesized within this model: hedonism (pleasure and play), aesthetics, symbolism (non-verbal communication), enactive transitional space, and generativity. Jones (2021) proposed the following core processes across the arts therapies: artistic projection, the triangular relationship, perspective and distance, embodiment, non-verbal experience, the playful space and the informed player, the participating artist-therapist, and the active witness.

### Change Process Research

In psychotherapy research, there has been growing interest in expanding the range of studies beyond mere outcome research. Whereas, psychotherapy outcome research inquires whether or not treatment leads to change, change process research (CPR) inquires *how* or *why* psychotherapy leads to change (e.g., Ramsayer and Tschacher, 2011). In the CATs, it is important for research, practice, and education to identify factors that lead to therapeutic change and that are associated with certain outcomes; however, many research funding bodies are almost exclusively interested in outcome research and their implications for policy. The importance of studying what unfolds and what is helpful in a single session or across several sessions has long been acknowledged by leading psychotherapy researchers (Timulak, 2008; Kazdin, 2009; Elliott, 2010; Lambert, 2013; Gelo et al., 2015). CPR is crucial to the advancement of the CATs, because it can help to: (a) identify specific therapeutic factors that can account for the ways in which therapeutic change occurs, (b) improve the effectiveness of CATs interventions, (c) refine a theory of change that provides a rationale and structure for CATs interventions, and (d) develop more effective training and supervision on effective therapeutic factors that are supported by evidence (Hardy and Llewelyn, 2015).

Data in CPR can be collected from one or, preferably, several perspectives including the client, therapists, and observers; change can stem from the client, therapist, or relational processes (Elliott, 2012). Type of change may be cognitive, behavioral, emotional, and/or physiological; the locus of change may be at the individual, interpersonal, and/or community/socio-cultural levels. These somewhat artificial divisions can be helpful “as a starting point for thinking about what initial position [and theory] a particular therapy adopts regarding change” (Dallos and Vetere, 2005, p. 98).

The collection of process data is routinely done using a variety of both qualitative and quantitative methods. Elliott (2010) identified four types of CPR. The first type is qualitative helpful factors research that examines which factors (i.e., client, therapist, or relational process factors) lead to client-reported change as assessed post-session or post-treatment. The second is quantitative micro-analytic sequential process research that examines the associations between process factors themselves by coding clients' and therapists' recorded responses and interactions (e.g., how specific responses or techniques provided by the therapist are associated with client engagement or insight). This design is best suited to quantitatively testing theory of change hypotheses. The third is significant events research that examines what happens in client-identified important moments in therapy (helpful or hindering) and may involve both qualitative and quantitative data collection and analyses. The fourth type is quantitative process-outcome research, which involves measuring process variables and testing whether they relate to or influence therapeutic outcomes (see also Crits-Christoph et al., 2013; Hayes, 2013). Process variables are often labeled as *therapeutic factors* or *change factors*, as well as *mechanisms of change* or *mechanisms of action*, as described next.

### From Common and Specific Factors to Mechanisms of Change in Psychotherapy Research

The literature on psychotherapy research employs terms that are used interchangeably by researchers across treatment contexts, from medicine to psychotherapy. This section aims to elucidate the range of terms and suggest definitions pertinent to CATs (see Table 2).

**Table 2:** Definition of terms.

**Term**	**Definition**
Common factor	A therapeutic/change factor that is common to all psychotherapy approaches. Also termed non-specific factor or universal factor (a-theoretical).
Specific factor	A well-specified therapeutic/change factor that is theorized to produce therapeutic benefits in a specific psychotherapy approach.
Joint factor	In this report, a therapeutic/change factor that is shared across the CATs disciplines.
Mechanism of change	A theory-driven causal chain or sequence of events or processes (or mediating variables) that explain, in greater detail than factors or mediators alone, how or why therapeutic change occurs.
Mediator	An intervening variable that is theorized to account for the statistical causal relationship between two variables, such that X causes M which in turn causes Y. A mediator clarifies how or why therapy leads to change and is a term usually used in the context of quantitative statistical analysis.
Moderator	A variable external to the treatment that influences the direction or magnitude of the statistical relationship between the treatment and outcome, such that a moderator may strengthen, weaken, diminish, or reverse the relationship between X and Y. A moderator clarifies when or for whom therapy leads to change and is a term usually used in the context of quantitative statistical analysis.

*Partly based on Kazdin (2009)*.

Many comparative studies have reported equivalent outcomes across diverse psychotherapies, which has been dubbed the “Dodo effect” (Rosenzweig, 1936). When all psychotherapy methods are found to have equally beneficial effects, “everyone wins the race, and all get a prize,” according to the verdict of the Dodo bird in the book *Alice in Wonderland* (Carroll, 1971/1865). This effect has been attributed to: (a) methodological problems (e.g., correlational design and low statistical power due to small sample size), (b) the notion that different psychotherapies may lead to comparable outcomes through different pathways, and (c) the notion that therapeutic change owes more to common factors than to specific factors in a particular psychotherapy approach (Grawe, 1997; Lambert, 2013).

Over the last few decades, psychotherapy researchers have put forward several models or taxonomies of *common factors* (also termed non-specific factors or universal factors), defined as “elements common to all psychotherapeutic approaches” (De Felice et al., 2019, p. 50). Reviewing these models in full is beyond the scope of this article (see Lambert, 2013; Cuijpers et al., 2019), but the common factors that are frequently reported include client involvement, client expectations of outcome and perceived treatment credibility, self-understanding, and insight, as well as the therapeutic alliance (i.e., positive client-therapist relationship). The latter is the most frequently researched common factor that can be measured by self-reports by both client and therapist (Horvath and Greenberg, 1989), as well as by observational coding (McLeod and Weisz, 2005). Essentially, common factors are a-theoretical, in that they are broad and not rooted in a specific theory of change (Wampold, 2001, 2015). Rather, they are related to client characteristics, therapist characteristics, and their interactions (Lambert, 2013). Table 3 displays a list of the main common factors in the general psychotherapy literature (e.g., Grawe, 1997; Norcross, 2011; Lambert, 2013; Wampold, 2015; Wampold and Irmel, 2015).

**Table 3:** Common factors of psychotherapy.

**Therapist**	**Client**	**Interaction**	**Extra-therapeutic**
Empathy	Motivation	Therapeutic relationship (e.g., alliance, bond, rapport, goals, and tasks agreement)	Environment
Warmth	Expectations of therapeutic success	Real relationship (i.e., genuine relationship that is transference-free)	Support system
Positive regard/affirmation	Believed credibility of treatment	Synchrony	Life events
Congruence/genuineness	Trust/safety	Goal consensus	Community
Respect	Agency (i.e., client as generator of change)	Collaboration/cooperation	Socioeconomic status
Acceptance	Involvement/engagement	Expressive attunement (the quality of communication)	Client experiences between sessions
Feedback to client	In-session behavior (e.g., emotional, cognitive, behavioral exploration, resistance)	Affective attitude (feelings of client and therapist toward each other)	
Therapist individual characteristics	Hopefulness about treatment	Work in the here-and-now	
	Self-understanding		
	Learning		
	Insight		
	Emotional release (abreaction)		
	Release of tension		
	Experiencing level		
	Corrective emotional experience		
	Client feedback to therapist		
	Mastery of/control over/coping with the problem		
	Problem clarification/meaning		
	Problem actualization		
	Resources activation (e.g., strengths, abilities)		

*In group therapy, Yalom (1995) has identified 11 common therapeutic factors: altruism, cohesion, universality, interpersonal learning input and output, guidance, catharsis, identification, family re-enactment, self-understanding, instillation of hope, and existential factors*.

*Specific factors* (also called specific ingredients) are well-specified therapeutic factors that are theorized to produce therapeutic benefits in a specific psychotherapy approach. These include, for example, interpretation in dynamic psychotherapy, emotional arousal and processing in experiential therapy, cognitive restructuring in cognitive therapy, and behavioral modification in behavioral therapy, and so forth (Crits-Christoph et al., 2013). In the study of specific factors, it is particularly important to monitor the therapist's *adherence* to the treatment protocol and/or to approach-specific techniques as well as the level of *competence* (i.e., skillfulness) in which the treatment is delivered (Wampold, 2015).

However, while some researchers proposed that at least 80% of the variance in outcomes are attributed to client or extra-therapeutic factors as well as unexplained and error variance, others have argued that this is a misconception, because common and specific factors are significantly correlated and thus cannot be considered independently (De Felice et al., 2019). For example, a technique in a specific approach (specific factor) can be helpful because there is a strong alliance between the therapist and the client or because the client has high expectation or motivation for change (common factors). Consistently, researchers were called to consider how common and specific factors can be “integrated or synthesized in a meaningful change process framework” (van der Merwe, 2020, p. 77).

Finally, the terms factors, ingredients, mediators, and mechanisms of change or mechanisms of action are used in the literature interchangeably to describe what leads to or causes therapeutic change in an outcome of interest. We view factors and ingredients as two overlapping terms, with the former being more prevalent in the psychotherapy literature and the latter in medical and pharmaceutical literature. For example, as mentioned above, psychotherapy research addresses therapeutic factors that impact a target outcome of interest, such as changes of maladaptive thoughts impact depression in cognitive therapy (Kazdin, 2009). Comparably, medical pharmaceutical research addresses active ingredients in a given medicine that has therapeutic effects on the body, such as increased levels of serotonin in the brain through antidepressant medicine based on selective serotonin reuptake inhibitors (SSRI; Preuss et al., 2020).

### The Present Study

Since previous systematic reviews have mainly focused on the impact of the effects of CATs interventions on certain outcomes (e.g., Van Lith, 2016; Koch et al., 2019; Orkibi and Feniger-Schaal, 2019; De Witte et al., 2020b; Feniger-Schaal and Orkibi, 2020), it is worthwhile gaining more insights into how, why, and when CATs interventions lead to certain outcomes. The present study consists of a scoping review of the literature followed by an in-depth analysis of the therapeutic factors and mechanisms of change. The objectives of this study were: (a) to clarify key concepts and definitions, (b) to identify the evidence from empirical studies on CATs therapeutic factors and mechanisms of change, (c) to identify *joint factors* across the CATs disciplines, and *specific factors* that are unique to each CATs discipline (see **Figure 2**) as opposed to common factors across psychotherapies, (d) to examine how research is conducted on CATs therapeutic factors and mechanisms of change, (e) to establish a framework for future studies on CATs mechanisms of change, and (f) to identify, analyze, and discuss gaps in knowledge.

## Methods

### Scoping Review Design

We conducted a scoping review to identify the therapeutic factors and mechanisms of change of the CATs reported in empirical studies. Scoping reviews are generally used to map the concepts underpinning research and the main types of evidence (Arksey and O'Malley, 2005), and thus differ from systematic reviews, which are mainly used to address more specific questions based on particular criteria of interest (Peters et al., 2015). Scoping reviews can be seen as a hypothesis-generating exercise, while systematic reviews often focus on hypothesis-testing (Tricco et al., 2016). Like systematic reviews, scoping reviews also require comprehensive and structured searches of the literature to maximize the relevant information, provide reproducible results, and decrease potential bias from flawed implementations (Sucharew and Macaluso, 2019). The results of a scoping review can provide in-depth information for further orientation, define preliminary working hypotheses, set research agendas, and identify implications for decision-making (Tricco et al., 2016; Von Elm et al., 2019). Scoping review methods are often used to bundle research results and to identify gaps as well as recommendations for further research (Munn et al., 2018). Therefore, a scoping review was the best fit for our purposes.

### Expert Group

To effectively implement all the steps of this scoping review, and to guarantee the global perspective we set out to retain, we formed an international multidisciplinary expert group. This expert group consisted of 10 CATs researchers from different countries and universities. The team included researchers who are experts in art therapy (1), drama therapy and psychodrama (2), music therapy (3), dance movement therapy (3). In close consultation and discussion with each other, the experts came to a consensus on the scope and methods of this review. The first task was to agree on the definition and related terms of the key concepts “therapeutic factors” and “mechanisms of change.” Terms were extracted from literature and synthesized by three expert group members, after which working definitions were created. Terms and definitions were then discussed in the full expert group to further refine them and to reach a consensus, which finally resulted in a shortlist of potentially relevant sub-terms for each term (see Table 2 for term definitions). The second task of the expert group was to formulate appropriate inclusion and exclusion criteria for selection of the articles. The diversity in both study designs and the content of the different types of CATs interventions required an approach that, despite the formulated criteria, allowed for individual considerations with respect to each study.

### Search Strategy

#### Inclusion Criteria

Multiple inclusion criteria were formulated in consultation with all the experts. First, only empirical studies (i.e., consisting of data collection methods) published in peer-reviewed scientific journals, reporting on therapeutic factors and/or mechanisms of change of CATs interventions, were included. Second, we only included articles written in English. Third, an important inclusion criterion was that the study was explicitly situated within a CATs context; in other words, for example, that the CATs intervention was provided by a certified creative arts therapist. Fourth, both quantitative and qualitative data-driven studies as well as systematic literature reviews were included; i.e., that the therapeutic factors and mechanisms of change were derived from empirical data. Case studies were included only when they were data-driven and consisted of empirical methods of data collection. Fifth, because this scoping review focused solely on psychological well-being outcomes, studies that dealt exclusively with medical or physical outcomes were excluded. Finally, no restrictions were placed on client populations so that a wide range of health conditions, gender, culture, context of adversity, and age groups (children, adults, elderly) were included.

#### Databases and Search Terms

Using a pre-defined search strategy, we systematically searched the literature for relevant articles. A computer-based search was conducted of the psychological and medical electronic literature databases without restriction on publication dates, including Medline, Academic Search Complete, Cochrane Library, Web of Science, Embase, Wiley Online Library, Springerlink, PubMed, PiCarta, Academic Search Premier, ScienceDirect, PsycINFO, and Google Scholar. Appropriate search terms were identified based on the findings of the expert group on how “therapeutic factors” and “mechanisms of change” and related terms were defined within the literature. The search strategy included six sets of search terms: those that (i) apply to “mechanisms of change/therapeutic factors,” are relevant to (ii) “creative arts therapies” as an umbrella term, (iii) “art therapy,” (iv) “dance movement therapy,” (v) “drama therapy” or “psychodrama,” (vi) “music therapy.” For all the searches conducted, search terms related to “mechanisms of change/therapeutic factors” were combined with terms referring to “creative arts therapies” or to one of the specific CAT disciplines. See Supplementary Materials for an exemplary search string that was used for the PsycINFO database.

#### Selection of the Articles

**Figure 1:**
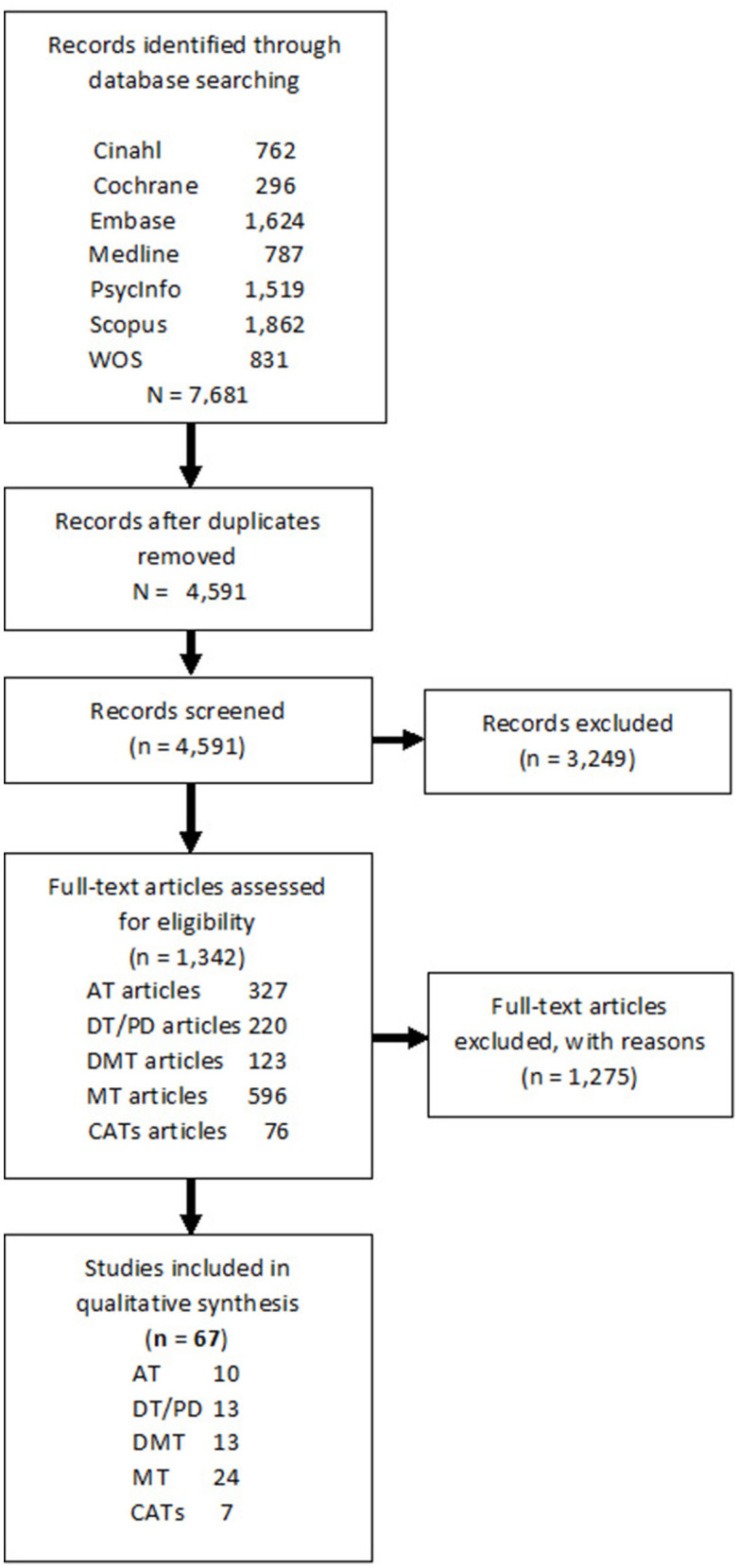
Flow chart of the search.

The initial search was conducted by an independent medical librarian and the first and second authors and adhered to the search criteria approved by all the co-authors. All the publications available up to January 2020 that met the inclusion criteria were uploaded to Rayyan QCRI^1^, which is a widely used online workspace for screening articles systematically where each screener is blind to the other screeners' selections. This resulted in a total of 4,591 publications, which were then screened in two separate selection steps. All the steps of the selection process were conducted by at least two authors, who selected the studies independently of each other. In cases of selection conflicts, a third author made the final decision. The first selection step, based on title and abstract, resulted in 1,466 publications. The selected publications were labeled according to their specific CATs discipline. In the second selection step, based on full text publications, separate Rayyan workspaces were created for each specific CATs discipline, and for the publications that consisted of more than one CATs discipline. In each workspace, publications were fully screened based on the inclusion criteria by at least two authors specialized in the particular CATs discipline. In addition to the electronic search in the online databases, 11 articles were included based on hand searches of the reference lists in the articles and consultation with experts. In total, 67 articles met all the inclusion criteria for this scoping review, as shown in Figure 1.

### Coding and Data Analysis

#### Coding of the Studies

To report on the possible and/or assumed therapeutic factors and mechanisms of change in the CATs studies, several characteristics of the included articles were identified and coded independently by two authors using a coding Excel sheet, and conflicts were discussed until an agreement was achieved. In terms of the study characteristics, we coded the design, the type of setting, and context of the study. First, we separated the quantitative studies from the qualitative studies, because the methodology required a different form of coding. The quantitative designs covered randomized controlled trials (RCT), clinical controlled trials (CCT, without randomization), pre-post designs (one group), systematic reviews (SR), or meta-analyses. For each of the quantitative studies, we coded the targeted outcomes, the type of measures used, and the main results. For the qualitative study designs, we coded whether the study used focus groups, interviews, clinical observations, or data from self-report measures. We also coded the research purpose and the main findings of each qualitative study. The type of setting refers to the specific context in which the study was conducted (e.g., schools, forensic psychiatry, palliative care, outpatient- or clinical care). We also coded the target group or client group involved (e.g., children/youth/adults/older adults, inmates, clients suffering from depression or anxiety, older adults suffering from dementia, etc.). We also coded in which country the study was conducted, the frequency and number of therapy sessions, and whether there were group or individual sessions. For the specific characteristics of the studies in each CATs discipline, see Supplementary Tables 1–5.

#### Data Analysis

**Figure 2:**
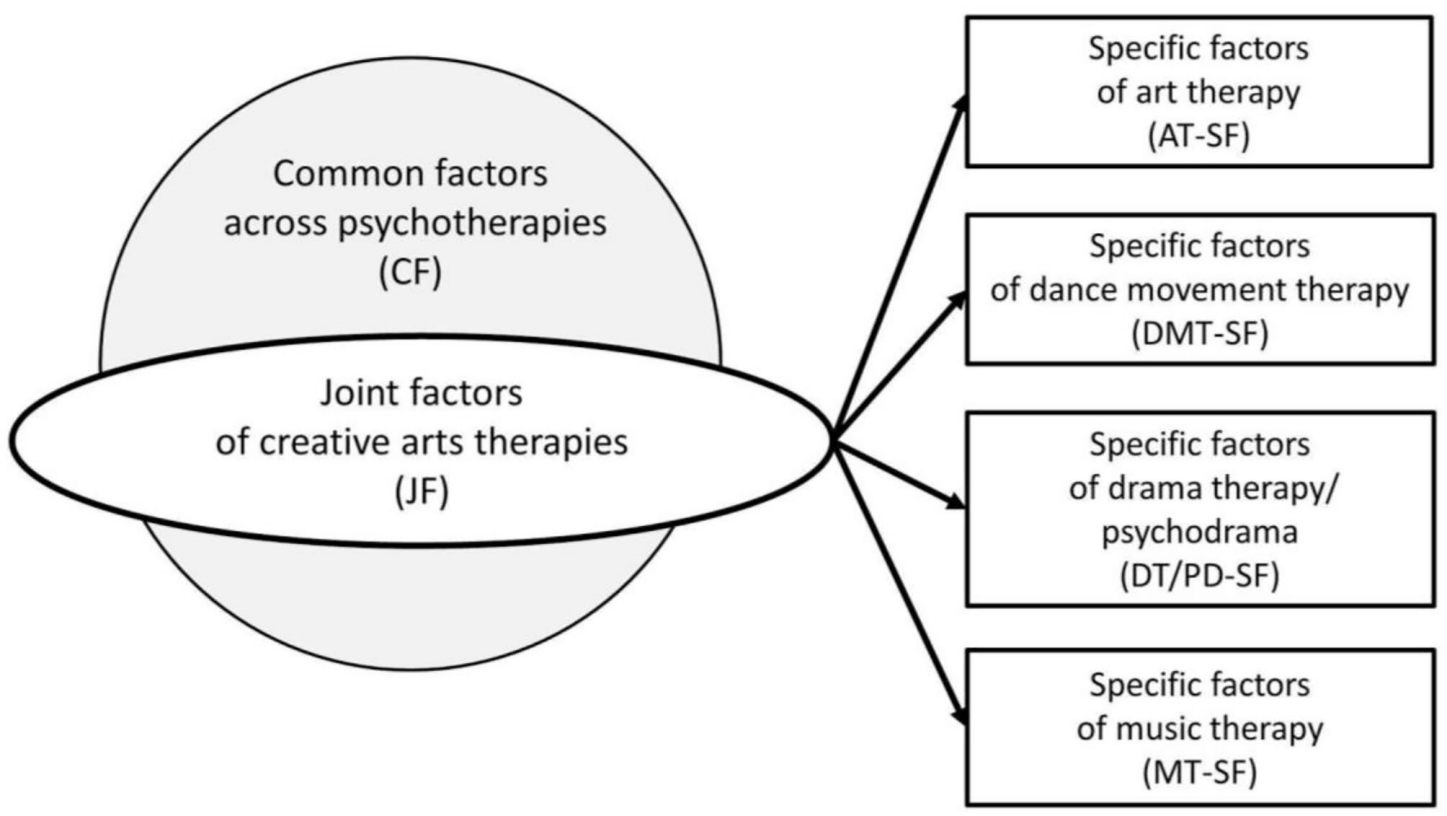
Types of therapeutic factors.

The first coding step provided initial insights into the articles. Next, we specifically focused on the relationship between the intervention characteristics, therapeutic factors or mechanisms of change, and the effects or targeted outcomes reported in each of the studies. To provide more insights into which of the CATs factors were similar or different from the common factors of psychotherapy, we further analyzed each of the therapeutic factors. First, we coded the therapeutic factors as a *common factor* (CF) when there was a strong association with the psychotherapeutic factors compiled from the literature on psychotherapeutic common factors (see Table 3). The next step was to identify which of the therapeutic factors that remained could be regarded as a *specific factor* (SF); i.e., the therapeutic factor was specific to one discipline of the CATs, rather than shared by the CATs disciplines. Therapeutic factors that appeared in more than one CATs discipline, and were not CF of psychotherapy, were therefore regarded as shared factors which we coded as *joint factors* across the CATs (JF); see Figure 2.

**Figure 3:**
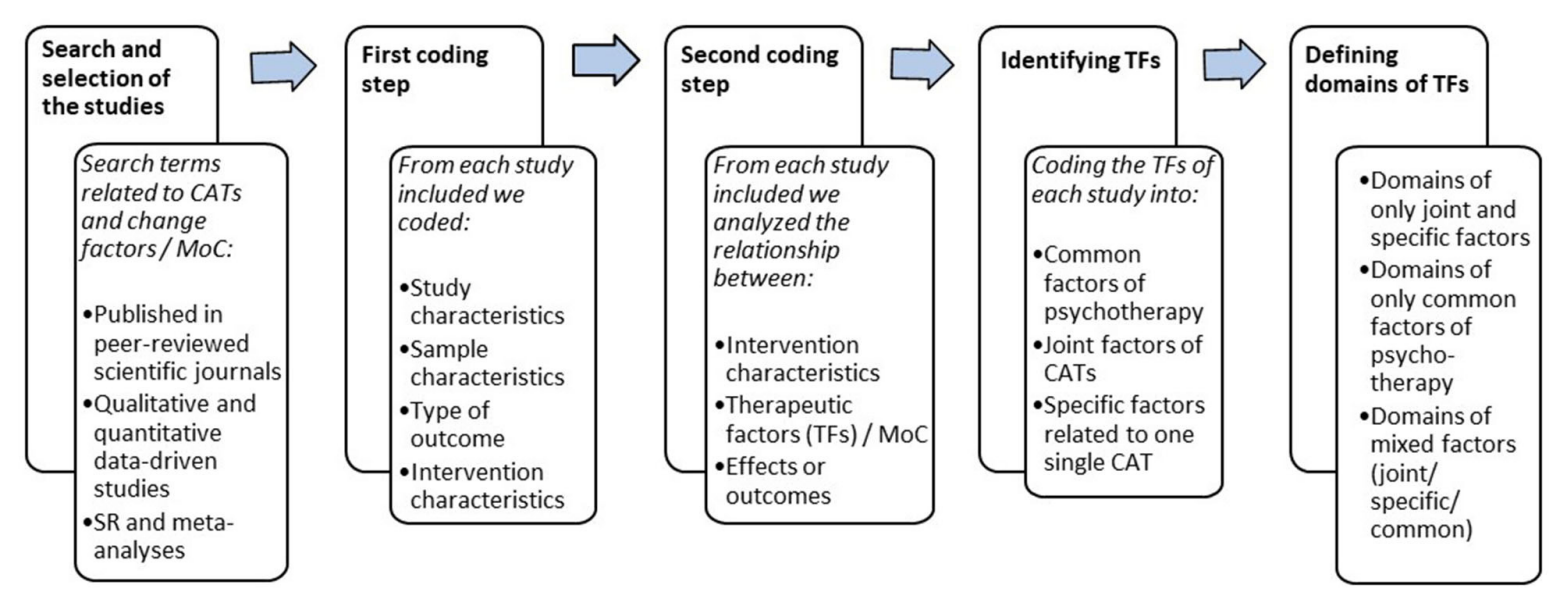
Overview of the steps of data collection and analysis.

To provide insights into the relationship between all the therapeutic factors from a broader perspective, all the therapeutic factors were categorized by three members of the expert group by inductive analysis. This approach is used in many qualitative data analyses and aims to gain an understanding of meaning in complex data through the development of summary themes or categories from the raw data (e.g., Backett and Davison, 1995; Thomas, 2003). The inductive approach allows research findings to emerge from the frequent, dominant, or significant themes inherent in the raw data, without the restraints imposed by structured methodologies (Thomas, 2006). Starting with the JFs, the inductive analysis further reduced the data into several main categories and corresponding subcategories of therapeutic factors. The second step involved adding the CFs and SFs to these main categories, which resulted in splitting these categories or expanding them with new subcategories. The data analysis process was carried out in its entirety by three researchers and consisted of a continuous iterative process in which all decisions were made by consensus. After the analysis was completed, the whole process was verified by one co-author who was not actively involved in the data analysis. To finalize the results of our analysis we determined whether each domain subcategory was made up of only SFs and JFs of the CATs or only the CF of psychotherapy, or whether it consisted of a mixed therapeutic factor. For a complete overview of the steps taken concerning the data collection and analysis, see Figure 3.

## Results

Of the 67 studies included in this scoping review, 28 were quantitative, 34 were qualitative, and five used mixed methods designs. Of these, one was a theoretical review, two were narrative reviews, six were systematic reviews, and 58 were original studies that specifically addressed therapeutic factors and/or mechanisms of change in the CATs in a wide variety of contexts. In the following paragraphs, we examine the studies reviewed in each specific discipline in terms of the *common factors* of psychotherapy (CF), *joint factors* of therapeutic change across the CATs (JF), and *specific factors* of drama-, dance-, art-, and music-therapy (SF). Note that the findings are presented with the original wording used by the authors to describe therapeutic factors and/or mechanisms of change (see Supplementary Tables 1–5). We conclude with an overview of 19 domains of therapeutic factors that were generated by further analysis (see Table 4).

**Table 4:** The 19 domains of therapeutic factors in the CATs.

**Domain of TF**	**CATs factors (joint and specific)**	**Mixed-type factors (joint, specific, and common)**	**Common Factors**
**Embodiment (*****n*** **=** **14)**	• ***Embodiment*** • Embodiment (DT/PD-JF, AT-JF, DT/PD-SF) • ***Body awareness*** • Body awareness (AT-JF) • Kinesthetic awareness (DMT-JF) • Body-mind connection (DMT-SF) • Body self-perception (DMT-SF) • Getting in touch with the body (DMT-SF) • ***Physicality of the arts*** • Physical experience with the body (DT/PD-JF) • Tactile quality (AT-SF) • Enactment (DMT-SF) • Physical act of music making (MT-SF) • Altering of inherent body-rhythms (MT-SF) • ***Experiencing the body*** • Experiencing the body (DMT-SF)		
**Concretization (*****n*** **=** **10)**	• ***General*** • Creating something visible (DT/PD-JF) • Artistic product facilitates verbal reflection and self-knowledge (CAT-JF) • Re-enactment (DMT-JF) • Self-display (DMT-JF) • Performing biographic themes (DMT-JF) • Designing dynamic themes (DMT-JF) • Role reconstruction (DT/PD-SF) • Changing and embodying of roles (DMT-SF) • Visual narrative of life (AT-SF) • Portraying feelings of past/future (AT-SF)		
**Symbolism and metaphor (*****n*** **=** **8)**	• ***Symbolic work*** • Enhancing symbolic elaboration (CAT-JF) • Shifting between symbolic and knowing realm (DMT-JF) • Metaphor (CAT-JF) • ***Unconscious processes via arts*** • Unconscious self-expression (AT-JF) • Interpreted symbols and images (MT-JF) • Transference to the artistic product (AT-SF) • Dramatic projection (DT/PD-SF) • Movement metaphor (DMT-SF)		
**Agency (*****n*** **=** **24)**	• ***Artistic agency*** • Artistic agency (MT-JF) • Agency in music making (MT-SF) • Jumping rhythm (DMT-SF) • Offering music choices (MT-SF) • Discovering materials and possibilities (AT-SF)	• ***General agency*** • Enhanced self-concept (CAT-JF) • Activating self-agency (DMT-JF) • Offering control and choice (DT/PD-JF) • Ownership (MT-CF) • Strengthened agency (CAT-CF) • Empowerment (CAT-CF, DT/PD-CF) • Empowering experiences (MT-CF) • Productive behaviors (DT/PD-CF) • Self-efficacy (DMT-CF) • Agency (MT-CF x2) • Enhancement of self-esteem (DMT-CF) • Developing sense of achievement (AT-CF) • ***Motivation*** • Vitality/vitalization (DMT-JF x2) • Music as motivating (MT-CF) • Motivational force when unwell (AT-CF) • Impacts present energy level (MT-CF)	
**Interaction through the arts (*****n*** **=** **19)**	• ***Empathy*** • Non-verbal attunement (MT-JFCAT) • Musical attunement (MT-SF) • Mirroring and movement (DMT-SF) • Doubling (DT/PD-SF) • Role reversal (DT/PD-SF) • ***Dialogue*** • Interacting with one another in movement (DMT-SF) • Intramusical connections (MT-SF) • Musical dialogue (MT-SF) • Shared musical experiences (MT-SF) • Turn taking (MT-CF) • ***Complex interactions*** • Relational aesthetic (AT-JFCAT) • Transference during artmaking (AT-SF) • Triggering musical encounters (MT-SF)	• ***Synchronicity*** • Therapist working alongside the client within and outside of the drama (DT/PD-JFCAT) • Moving in synchronicity (DMT-SF) • Musical synchronicity (MT-SF) • Joint attention (MT-CF) • Moving with the therapist (DMT-SF) • Modeling (MT-CF)	
**Structure (*****n*** **=** **17)**	• ***Structure of the art form*** • Structuring nature of music (MT-SF x2) • Structuring/repetitive nature of music (MT-SF x2) • Structuring/safe nature of music (MT-SF) • Repetitive rhythm (MT-SF) • Tempo of the music (MT-SF) • Slow and steady music tempo (MT-SF) • Musical simplicity (MT-SF)	• ***Structure of session*** • Structuring emotional outlets (DMT-JF) • Implementing structure (MT-JF) • Rituals (DMT-JF, AT-JF) • Programmed classical music (MT-SF) • Tailored structure/content to fit the client (CAT-CF) • Bringing it all together (CAT-CF) • Predictability (MT-CF)	
**Developing skills (*****n*** **=** **16)**	• ***Artistic skills*** • Developing artistic talents (CAT-JF) • To learn or practice artistic skills (AT-JF) • Learning new ways of living in the body (DMT-SF) • Connecting movement and language with one another (DMT-SF) • Learning and practicing of motion sequences (DMT-SF) • Musical expansion (MT-SF) • Enhancement of control through musical improvisation (CAT-SF) • Enhancement of concentration through music improvisation (CAT-SF)	• ***Personal and interpersonal skills*** • Skills training (DMT-JF) • Improving feedback skills (AT-CF) • Prosocial behavior (MT-CF) • Verbal and non-verbal skills (MT-CF) • Developing mastery in processing/communication of emotions (CAT-CF) • Enhancing emotional intelligence (DMT-CF) • Mastery of dynamic challenges (DMT-CF) • Increased social skills and connection (CAT-CF)	
**Active engagement** **(*****n*** **=** **15)**	• ***Engagement with artistic activity*** • Dramatic engagement (DT/PT-SF) • Therapeutic activity (CAT-JF) • Mobilization (DMT-JF) • Engagement with the musical experience (MT-SF) • Musical engagement (MT-SF) • Use of specific art materials and techniques (AT-SF)	• ***General involvement*** • Encouraging active engagement (CAT-JF) • Active engagement (MT-JF) • Being actively involved (DT/PD-JF) • Being actively involved in therapy (DT/PD-JF) • Client involvement (DT/PD-JF) • Client targets (CAT-CF) • Personal responsibility (CAT-CF) • Client in-session behaviors (DT/PT-CF) • Experiencing level (DT/PD-CF)	
**Creativity (*****n*** **=** **13)**	• ***General*** • Creativity (CAT-JF) • Creative self-expression (MT-JF) • ***Spontaneity*** • Spontaneity (DT/PD-JF) • Moving spontaneously (DMT-SF) • ***Experimentation*** • Trying out new ways of being (DT-JF) • Opportunity to explore (AT-JF) • Designing and testing (DMT-JF) • Artmaking in session as a form of exploration (AT-SF)	• ***Letting go of control*** • Losing control (DMT-JF) • Loosening up of movement (DMT-SF) • Mobilizing/loosening-up in movement/making flexible (DMT-SF) • Non-goal orientation of dance and movement (DMT-SF) • Resistance decrease (DT/PD-CF)	
**Artistic pleasure (*****n*** **=** **13)**	• ***Playfulness*** • Playfulness (DT/PD-JF) • Play (AT-JF) • Playfulness involved in playing/sharing instruments (MT-SF X 2) • ***Aesthetics*** • Experience of beauty (DMT-JF)	• ***Pleasant feelings*** • Pleasure (AT-JF) • Aesthetic pleasure (CAT-JF) • Pleasure of movement (DMT-JF) • Pleasure from play (MT-JF) • Experiencing positive affect (CAT-JF) • Enjoyment (MT-JF x2) • Hope and optimism (DT/PD-CF)	
**Modulating time and space (*****n*** **=** **14)**	• ***Flow experience*** • Flow state (AT- JF) • Experience of flow (MT-JF) • Transcending thinking of product/process (AT-JF)	• ***Here and now*** • Surplus reality (DT/PD-SF) • Point of focus/link to the present (MT-CF) • Being in the present moment (MT-CF) • Working with the here and now (CAT-CF, DT/PD-CF) • Presence in the moment (CAT-CF) • Experiencing the present moment (AT-CF) • ***Distraction*** • Distraction of stress-increasing thoughts (MT-JF) • Temporary distraction from illness (MT-CF)	• ***There and then*** • Getting to the root (CAT-CF) • Facing deeper issue from the past (DMT-CF)
**Group processes (*****n*** **=** **25)**	• ***Encounter*** • PD “encounter” between group members (DT/PD-SF) • Encountering one another (DMT-SF)		• ***General*** • Group process (AT-CF) • Community engagement (CAT-CF) • ***Yalom's therapeutic factors*** • Yalom's therapeutic factors for group therapy (DT/PD-CF x4). • Altruism (MT-CF) • Instillation of hope (MT-CF) • Interpersonal learning (MT-CF) • Validating feedback of group members (MT-CF) • ***Group cohesiveness*** • Group cohesiveness (MT-CF x8) • Establishing cooperation and (group) cohesion (DMT-CF) • Connecting to others (DMT-CF x2) • Feelings of togetherness and bonding (MT-CF) • Acceptance of self/others (AT-CF)
**Non-verbal expression** **(*****n*** **=** **11)**		• ***General*** • Expression (MT-JF, AT-CF) • Non-verbal expression (CAT-JF, DMT-JF, AT-JF x2) • Expressivity in movement (DMT-SF) • Form of visual self-expression (AT-SF) • Use of tone (MT-SF) • Expression of emotion (DMT-CF x2)	
**Connection with self** **(*****n*** **=** **12)**		• ***General*** • Focusing to oneself (DMT-JF) • Connection to inner self (AT-CF) • Connecting to self (DMT-CF x2) • Self-exploration (DMT-CF) • Establishing inner connections (DMT-CF) • Finding inner balance (DMT-CF) • Strengthen sense of self (MT-CF) • ***Connection with self through the arts*** • Acceptance of artwork (AT-JF) • Portraying self-image (AT-SF) • Pride (MT-CF) • Personal values (MT-CF)	
**Remembering (*****n*** **=** **5)**		• ***General*** • Music stimulated autobiographical recall (MT-SF) • Reminiscence (MT-CF) • Stimulation of memory (CAT-CF) • Remembering (DMT-CF) • Reconnect with key moments (MT-CF)	
**Therapeutic alliance and bond (*****n*** **=** **10)**		• ***General*** • Therapeutic alliance (AT-CF, MT-CF, DMT-CF) • Therapist-client bond (DT/PD-CF, AT-CF) • Therapeutic relationships (MT-CF) • Fundamental relational skills and features (CAT-CF) • Developing supportive relationships (CAT-CF) • Receiving and providing support (DT/PD-CF) • Verbal dialogue (MT-CF)	
**Emotional elicitation and processing (*****n*** **=** **23)**		• ***Release and relief*** • Deep relaxation (MT-JF, AT-JF) • Release of intrapsychic tension through improvisation (CAT-SF) • Energy discharge/tension release (DMT-SF) • Catharsis (DT/PD-SF, MT-CF) • Releasing and relief (DT/PD-CF) • ***Confronting*** • Confronting oneself with emotions (DMT-JF) • Confronting oneself with own actions (DMT-JF) • Emotional reaction to materials (AT-SF) • Seeing own emotions through visual art (AT-SF) • Confronting (DMT-CF) • ***Emotional regulation*** • Working within a safe distance within or outside the drama (DT/PD-SF) • Musical cues that are used to ground and modulate distress (MT-SF) • Regulating emotions (AT-CF, DMT-CF) • Acting out and “living through” emotions and directing this process (AT-CF)	• ***Expansion of emotions*** • Broaden-and-build affect (DMT-CF) • Broaden-and-build *via* experience of positive emotions (DMT-CF) • Enhancing emotional well-being (DMT-CF) • Actualizing emotions (DMT-CF) • ***Processing emotions*** • Transforming emotions (DMT-CF) • Emotion processing (AT-CF)
**Understanding (*****n*** **=** **24)**		• ***Reflection*** • Reflection upon art (AT-SF) • Artmaking in session as a form of reflection (AT-SF) • Increased reflective functioning (DT/PD-CF) • Reflection on own patterns (AT-CF) • Witnessing (DT/PD-CF, CAT-CF) • ***Self-awareness*** • Self-awareness through artwork (AT-SF) • Awareness of ego function/self-realization (CAT-CF) • Self-awareness (DT/PD-CF) • Awareness/exploring own feelings (AT-CF) • ***Meaning-making*** • Shifting between movement and meaning (DMT-SF) • Meaning making (CAT-CF) • High levels of meaningfulness (MT-CF) • Meaning (MT-CF) • ***Gaining insight*** • Using bodily sensation as a source of information (DMT-SF) • Understanding the pattern/insight (DT/PD-CF) • Insights in emotions (AT-CF)	• ***Perception*** • Perceiving emotions (DMT-CF) • Perceiving own blockages (DMT-CF) • Metallization (DMT-CF) • Reframing (DMT-CF) • Reframing identity (MT-CF) • Differentiating/clarifying feelings/thoughts (AT-CF) • Cognitive regulation (AT-CF)
**Environment (*****n*** **=** **10)**			• ***General*** • Comfortable and liberating environment (CAT-CF) • Creating a safe place (DMT-CF) • Safe environment (AT-CF) • Safety (AT-CF, MT-CF, DT/PD-CF) • Psychological safe space (AT-CF) • Supportive /familiar atmosphere (MT-CF) • Levels of trust in healthcare providers and treatment plans (CAT-CF) • Positive and safe intervention for developmental stage for children and adolescents (AT-CF)

*In this table the 19 domains of therapeutic factors resulting from the analysis. The left column lists therapeutic factors that are unique to the CATs (SF of one CATs discipline and JF identified across the CATs). The middle column lists the mixed-type therapeutic factors (SF, JF and CF); The right column only consists of CF across psychotherapies*.

*The n is the number of therapeutic factors included in a given domain*.

*JF, joint factor across creative arts therapies; MT-JF, joint factor across creative arts therapies found in a music therapy study; AT-JF, joint factor across creative arts therapies found in an art therapy study; DMT-JF, joint factor across creative arts therapies found in a dance movement therapy study; DT/PD-JF, joint factor across creative arts therapies found in a drama therapy/psychodrama study*.

*CF, common factor of psychotherapy; MT-CF, common factor found in a music therapy study; AT-CF, common factor found in art therapy; DMT-CF, common factor found in dance movement therapy; DT/PD-CF, common factor found in drama therapy/psychodrama; CAT-CF, common factor found in a study with more than one CATs included*.

*SF, specific factor in a CAT discipline; MT-SF, music therapy specific factor; AT-SF, art therapy specific factor; DMT-SF, dance movement therapy specific factor; DT/PD-SF, drama therapy/psychodrama specific factor; CAT-SF, creative arts therapies studies specific factor*.

### Art Therapy Studies

There were 10 art therapy studies that met the inclusion criteria (see Supplementary Table 1), including two quantitative studies, six qualitative studies, and two systematic reviews.

#### Common Factors of Psychotherapy From AT Studies

Art therapy takes place in the safety of the studio *environment* (Van Lith, 2015; Abbing et al., 2018; Nolan, 2019; Bosgraaf et al., 2020) and functions in the context of a strong *therapeutic alliance* (Bosgraaf et al., 2020; Keidar et al., 2020). Art therapists work in the *here and now* (Haeyen et al., 2015) to facilitate the expression and *clarification* of thoughts and feelings visually (Deboys et al., 2017; Abbing et al., 2018; Czamanski-Cohen et al., 2019). Art therapy is *motivating* (Van Lith, 2015) and offers opportunities to deepen *understanding, insight* (Haeyen et al., 2015; Van Lith, 2015), and *mastery* in that art therapy encourages the development of cognitive and emotional skills (Haeyen et al., 2015; Van Lith, 2015; Abbing et al., 2018).

#### Joint Factors of CATs in AT Studies

Along with other CATs, art therapy provides a safe and *structured* pathway for playful, creative *experimentation* and *self-awareness* (Deboys et al., 2017; Gabel and Robb, 2017; Abbing et al., 2018; Nolan, 2019). Art therapy, like all CATs, *modulates one's sense of time and space;* art therapy promotes *relaxation* and a *flow state* (Abbing et al., 2018). Individuals and groups benefit from the opportunity to learn and practice *artistic skills and forms of visual self-expression* (Van Lith, 2015; Bosgraaf et al., 2020). The art-making process helps individuals and groups *symbolize* and externalize experiences that are not easily verbalized and *concretize* internal conflicts to facilitate perspective-taking and reflection (Deboys et al., 2017; Gabel and Robb, 2017; Abbing et al., 2018; Czamanski-Cohen et al., 2019; Bosgraaf et al., 2020). This *non-verbal expression* enables *emotional elicitation and processing* (Haeyen et al., 2015; Gabel and Robb, 2017).

#### Specific Factors of AT

The *tactile quality* of art (Abbing et al., 2018) and the *choice of appropriate and specific art materials* (Haeyen et al., 2015; Bosgraaf et al., 2020) contribute to reducing anxiety and enhancing self-concept. Researchers observe *transference during art-making and the artistic product* (Hilbuch et al., 2016) as well as the specific potency of *art-making* in promoting perspective taking and self-awareness. *Visual self-expression* and the creation of a *visual narrative* were demonstrated to enhance self-concept, self-esteem and promote emotion regulation (Bosgraaf et al., 2020).

### Dance Movement Therapy Studies

In DMT, 13 studies met the inclusion criteria (see Supplementary Table 2). Seven were quantitative studies, and five were qualitative studies, and one a narrative review.

#### Common Factors of Psychotherapy From DMT Studies

As expected, relational components were important therapeutic factors in DMT studies and were shared across other forms of psychotherapy. The *therapeutic alliance* (Shim et al., 2017) in one-to-one work and *connecting with others* (Shim et al., 2017, 2019) in groups were clearly present. Along with external connections, creating *connections with one's self* was also found to be an important agent of change (Shim et al., 2017, 2019), and finding one's inner balance was presented as another internal therapeutic factor (Mannheim et al., 2013). The DMT studies also highlighted the value of stress reduction (Ho et al., 2018), *release and relief* through expressing and actualizing emotions (Chyle et al., 2020), but also *expanding, regulating*, and *processing* emotions. For example, studies referred to the *broadening and building affect, enhancing emotional well-being* (Shim et al., 2019), *regulating and ultimately transforming emotions* (Chyle et al., 2020). *Perceiving* one's own emotions and blockages (Chyle et al., 2020), along with processes such as *reframing* (Shim et al., 2017, 2019) or *mentalization* (Shuper-Engelhard et al., 2019) were all thought to impact therapeutic change. As in other types of psychotherapy, enabling clients to *remember* important things (Chyle et al., 2020) and *facing deeper issues in their past* (Winther and Stelter, 2008) was reported to be therapeutic. Finally, the need for a safe *environment* was seen as an essential requirement for any type of therapy, and more so in DMT where the body and movement may elicit feelings of vulnerability. Creating a *safe place* was also reported as an important common factor of change within therapy (Shuper-Engelhard et al., 2019).

#### Joint Factors of CATs in DMT Studies

Common factors in DMT that were present across all the CATs included the discipline's capacity to engage clients in *artistic activity* and in this case, in movement, through *mobilization* (Shim et al., 2017, 2019; Chyle et al., 2020). Studies acknowledged that engagement with movement can provide *pleasure* (Mannheim et al., 2013), similar to *artistic pleasure* derived from engaging with other art forms. Some found that *aesthetics* were also linked to therapeutic change as well as the client's *experience of beauty* (Koch et al., 2016) is key. As is the case for other CATs, *creativity* and *letting go of control* (losing control according to Chyle et al., 2020) were regarded as an important first step that can allow for *experimentation* (designing of model situations and testing alternative behavior, Chyle et al., 2020) and for *symbolic work* to take place (shifting between the symbolic and the knowing realms, Ellis, 2001). DMT increases brain connectivity (Ventouras et al., 2015), and supports concretization, another important therapeutic process shared across CATs; in DMT this takes place through the direct use of the body and through the therapeutic factor of *self-display* (Chyle et al., 2020). The *re-enactment* of biographical or dynamic themes was another therapeutic factor under concretization (Chyle et al., 2020). The ways in which movement experiences are *structured* within sessions were also viewed as key, since they allow for participation in *ritualized processes* and the progressive *release of emotions* (Chyle et al., 2020); thus, supporting the *expression of emotions in a safe way*.

#### Specific Factors of DMT

*Embodiment* is an important factor of change in DMT practice. *Experiencing the body* (Chyle et al., 2020) and *body awareness* (Mannheim et al., 2013; Shim, 2015) are regarded as an important agent of change. Similarly, although verbalizations are not excluded, the *non-verbal nature* of DMT allows for *discharge of energy* and *release of tension* (Mannheim et al., 2013), *expressivity in movement* (Shim, 2015) and *non-verbal disclosure* (Chyle et al., 2020). This engagement with movement can communicate *unconscious processes* in the form of *movement metaphors* (Ellis, 2001) and support *insight* by relying on *bodily sensations as a source of information* (Chyle et al., 2020). It can also support the development of *artistic skills* such as *connecting movement and language* with one another or learning and *practicing motion sequences* (Chyle et al., 2020). *Artistic agency* is also facilitated and can be evident in the ways in which one moves. Koch et al. (2007), for example, showed that the *jumping rhythm* is presented as an important agent of change in clients with depression. Finally, the *therapeutic alliance* is encouraged through *kinesthetic empathy* in the form of *mirroring* (Shuper-Engelhard et al., 2019), *dialogue* in the form of *movement interactions* and *synchrony* in the form of *movement synchronicity* (Chyle et al., 2020).

### Drama Therapy and Psychodrama Studies

There were 13 drama therapy and psychodrama studies that met the inclusion criteria (see Supplementary Table 3). Of these, there were eight quantitative studies, two mixed method studies, one qualitative study, a systematic review, and a survey.

#### Common Factors of Psychotherapy From DT/PD Studies

Drama therapy and psychodrama employ common factors of psychotherapy such as the importance of establishing a safe *environment* and the value of a positive *therapeutic alliance* (Cassidy et al., 2014; Orkibi et al., 2017a). Drama therapists and psychodramatists employ techniques that facilitate *experiencing* in therapy, to develop a felt awareness (Armstrong et al., 2016). Drama therapy and psychodrama promote a sense of *agency* and empowerment (Bucută et al., 2018), *hope and optimism* (Bucută et al., 2018), *reflection* (Cassidy et al., 2017), *self-awarenes*s and *insight* (Bucută et al., 2018; Testoni et al., 2018) and facilitate emotional *release and relief* (Bucută et al., 2018). *Four* psychodrama studies examined the transformative potential of *group processes*, such as Yalom's common factors for group therapy, with a specific emphasis on catharsis (*emotional release*), insight, self-understanding, interpersonal learning, and the reciprocal process of giving and receiving support (Yalom, 1983, 1995; Kellermann, 1985, 1987; Oezbay et al., 1993; Kim, 2003; Testoni et al., 2018). Two studies dealt with the ways in which the observed changes were related to non-specific and productive *in-session client behaviors* such as cognitive, behavioral, and emotional exploration (Orkibi et al., 2017a,b) and one study highlighted the catalytic function of working in the *here and now* (Cassidy et al., 2014).

#### Joint Factors of CATs in DT/PD Studies

Drama therapy and psychodrama, like all CATs, elicit *active involvement* (Cassidy et al., 2014) within an *embodied* artistic process in which both verbal and *non-verbal expressions* are possible (Cassidy et al., 2017). Like other CATs, drama therapists and psychodramatists offer intrinsically *pleasurable* and *playful* (Orkibi et al., 2014) activities that promote a sense of *agency* in that participants experience control and choice, and creative *experimentation* where clients are invited to practice their spontaneity and try out new ways of being (Orkibi et al., 2014; Cassidy et al., 2017). Drama therapists and psychodramatists, like other CATs, employ *concretization*, the process of rendering internal experience visible and tangible, which further enables perspective-taking and insight (Cassidy et al., 2017). Witnessing, a *reflective process* common to all CATs, was also highlighted in one study (Orkibi et al., 2014).

#### Specific Factors of DT/PD

Drama therapists and psychodramatists facilitate change through active *engagement* with and within *dramatic* or *surplus reality* (Cassidy et al., 2014; Orkibi et al., 2014, 2017a). Emotional regulation and reflection are facilitated by *working at a safe distance* within or outside the drama (Cassidy et al., 2017). Drama therapists and psychodramatists encourage understanding, self-awareness, perspective, and empathy through *doubling* (Goldstein, 1971), *role-reconstruction* (Bucută et al., 2018), *encounter*, and *role-reversal* (Orkibi et al., 2014; Testoni et al., 2018). In addition, *dramatic embodiment* and *dramatic projection* were identified as two specific factors contributing to client experiencing a felt awareness, which facilitates change (Armstrong et al., 2016).

### Music Therapy Studies

There were 24 music therapy studies that met the inclusion criteria (see Supplementary Table 4). Of these, eight were quantitative studies, 11 were qualitative studies, three were systematic reviews, and one was a theoretical review.

#### Common Factors of Psychotherapy From MT Studies

The common psychotherapy factors in music therapy include working in the *here and now* (Ansdell et al., 2010; Carr et al., 2012) within a positive *therapeutic alliance* (Bonde, 2005; Kellet et al., 2019) in a *safe, predictable environment* (Robb, 2000; De Witte et al., 2020c). Several studies emphasized the importance of group processes such as *group cohesiveness* (Goldberg et al., 1988; Waldon, 2001; Ahonen-Eerikäinen et al., 2007; Carr et al., 2012; Dalton and Krout, 2015; Bibb and McFerran, 2018; De Witte et al., 2020a), *feelings of togetherness and bonding* (De Witte et al., 2020b), *altruism* (Gardstrom et al., 2017), *validating feedback* by others, and *interpersonal learning* (Goldberg et al., 1988; Gardstrom et al., 2017). Participants in music therapy experience a sense of personal connection (Rolvsjord, 2010; Dalton and Krout, 2015), pro-social *skills* (Warth et al., 2016), *meaning* (McDermott et al., 2013; Baker et al., 2015), *agency* (Ahonen-Eerikäinen et al., 2007; Potvin et al., 2018), *motivation* (Dalton and Krout, 2015; Gardstrom et al., 2017; Millet and Gooding, 2017), and *emotional release and relief* (Gardstrom et al., 2017). Client *in-session behavior* factors of involvement and engagement were represented in all studies.

#### Joint Factors of CATs in MT Studies

Music therapy, like all CATs, *actively engages* participants (Landis-Shack et al., 2017; Millet and Gooding, 2017) in a *creative* process (Rolvsjord, 2010) where *verbal, symbolic* (Bonde, 2005; Short et al., 2009; Baker et al., 2015; Bibb and McFerran, 2018), and *non-verbal expression* are possible (Ansdell et al., 2010). Music therapy heightens participants' sense of *artistic agency* (Potvin et al., 2018) and promotes emotional release and relief through *deep relaxation* (Short et al., 2009). Like all CATs, music therapy promotes well-being because it is *intrinsically enjoyable* (Ahonen-Eerikäinen et al., 2007; Gardstrom et al., 2017), and because it modulates one's sense of time and space, by bringing one into a state of *flow* (Baker et al., 2015) or as a *distraction* from stress-inducing thoughts (Porter et al., 2017).

#### Specific Factors of MT

The specific therapeutic factors in music therapy include the *physical act of music making* (Landis-Shack et al., 2017) and the *safe and structuring* (Robb, 2000) nature of music itself including the use of *tone, tempo, and alternating rhythms* (Ansdell et al., 2010; De Witte et al., 2020a,b,c). Music therapy enhances therapeutic alliance and group processes through *playful* (Passiali, 2012; Porter et al., 2017) *musical interactions* (Passiali, 2012; Bibb and McFerran, 2018), *shared musical experiences* (Porter et al., 2017)*, musical attunement (*McDermott et al., 2013)*, musical synchronicity* (Potvin et al., 2018), and *musical dialogue* (Kellet et al., 2019). Music therapy triggers or activates memories (McDermott et al., 2013; Porter et al., 2017; Bibb and McFerran, 2018) which, together with *intervention choices and musical cues* facilitate coping and emotion regulation (Robb, 2000; Baker et al., 2015; Landis-Shack et al., 2017).

### Studies With More Than One CATs Discipline

Seven studies with a number of CATs disciplines met the inclusion criteria (see Supplementary Table 5). Of these, there were two quantitative studies, two qualitative studies, and three reviews. As these are integrated studies, only the CF of psychotherapy and the JF across CATs are reported below.

#### Common Factors of Psychotherapy From Studies of CATs

These studies confirmed the impact of several common factors of change in psychotherapy beginning with the *therapeutic alliance* (Heynen et al., 2017). Their orientations differ in that some work in the *here and now* (Flanagan, 2004; Parsons et al., 2020), while others focus on past events (Parsons et al., 2020), creative arts therapists tailor the therapeutic goals to meet individual and group needs, facilitate fundamental relational skills and features, and promote supportive relationships (Heynen et al., 2017; Kalaf and Plante, 2019; Parsons et al., 2020). CATs take place in a liberating *environment* characterized by trust and safety (Chiang et al., 2019), in which clients can experience a sense of *agency* and *skill* with regard to processing and communicating emotions and forming connections with others (Dunphy et al., 2019; Kalaf and Plante, 2019). Creative arts therapists facilitate *self-awareness* and opportunities for *meaning-making* (Flanagan, 2004; Kalaf and Plante, 2019).

#### Joint Factors of CATs

These studies revealed that the CATs *actively engage* (Parsons et al., 2020) participants in artistic therapeutic activity and offer multiple options for *verbal* and *non-verbal expression* through *symbolism* and *metaphor* (Flanagan, 2004; Schiltz, 2014; Chiang et al., 2019; Dunphy et al., 2019). The CATs facilitate *concretization*, to *make visible internal conflicts*, which enables *perspective-taking* (Schiltz, 2014; Kalaf and Plante, 2019). In addition, participants experience a sense of *creativity, artistic pleasure*, and achievement as they develop *artistic skills* (Chiang et al., 2019; Dunphy et al., 2019; Parsons et al., 2020).

### Domains of Therapeutic Factors Across CATs Studies

Table 4 presents the 19 domains of therapeutic factors across the CATs that were generated by further analysis. Note that these domains are not mutually exclusive and some are interrelated. The left column in Table 4 indicates therapeutic factors that are unique to the CATs; i.e., SF relevant to one CATs disciplines (e.g., MT-SF) and JF identified across the CATs. The middle column lists the mixed therapeutic factors; i.e., those that are SF, JF, and CF across psychotherapies (e.g., DT/PD-CF). The right column only consists of CF across psychotherapies.

## Discussion

The therapeutic factors identified in the 67 studies converged to 19 domains. We distinguished the CF of psychotherapy from the JF of CATs and identified the SF of each CATs discipline.

### Domains of Therapeutic Factors Unique to CATs Research

Of the 19 domains of therapeutic factors, three appeared to be unique to the CATs because they consisted of only SFs and JFs: embodiment, concretization, symbolism, and metaphors. In *embodiment*, getting in touch with the body and achieving body awareness lead to a fuller experience of the body. The physicality of the body-mind music/art making connection with the arts in the form of the sensory quality of the materials, enactment, and the physical act of artistic creation were designated as important therapeutic factors across the CATs (Koch, 2017; Lange et al., 2018).

*Concretization* refers to changing an abstract content or statement into a tangible form that can be physically perceived, experienced, and related to. *Concretization* constitutes a core element in what Blatner (1992) termed the “dynamics of change,” and further clarified that “creating something visible, audio, re-enacting an event or reconstructing a role permits reflection and ultimately, insight and new perspectives” (p. 409). It was mentioned across the CATs mainly because they involve shaping abstract content or experiences into a tangible form. This specific therapeutic factor warrants further empirical investigation (in drama see, Kushnir and Orkibi, 2021).

Relatedly, the CATs involve the projection and expression of internal and often unconscious material into visual, embodied, musical, and enacted forms. *Symbolism* allows clients to explore difficult material, experience transference and create links between known and unknown realms (Ellis, 2001; Short et al., 2009; Gabel and Robb, 2017). Imagery and metaphors are processes closely linked with the capacity to symbolize and operate as therapeutic factors in CATs contexts (Flanagan, 2004). These factors were pinpointed by Karkou and Sanderson (2006) and align with how Blatner (1992) described the underlying principle and unique function of the arts as transitional objects that allow for the projection of emotions and ideas onto the art medium. Symbolism as a JF also aligns with Johnson's (1998) explanation of therapeutic action, where “inner states are externalized or projected into the arts media, transformed in health-promoting ways and then re-internalized by the client” (p. 85).

In addition to the domains that consist solely of SFs and JFs, we identified 14 domains consisting of mixed-type factors (both SF/JF of CATs and CF of psychotherapy), six of which we discuss in more detail, because they reflect the integration of CF with arts-related processes. For example, the domain of *agency* is closely related to motivation, self-efficacy, and a sense of vitality/vitalization. While not the primary purpose of the CATs, participants often derive a sense of competence and accomplishment related to having artistic choices, or “*artistic agency*;” for example, when singing in harmony (Ansdell et al., 2010), making smooth movement transitions (Koch, 2014), performing their story (Bucută et al., 2018), or developing the visual art technique of shading (Bosgraaf et al., 2020).

The *active engagement* through the arts in CATs gives clients the opportunity to experience interpersonal communication through non-verbal attunement in music, mirroring in movement, and role-playing in drama. It is often the case that therapists and clients are equally active in the artistic process and may experience an arts-based synchronicity specific to CATs, which is another therapeutic factor (Ramsayer and Tschacher, 2011; Feniger-Schaal et al., 2018). Engagement *via* the arts has also been referred to as relational aesthetics; namely, “the overlapping triangular relationship between group members, artworks, and leaders in which the art serves as a medium for visual/non-verbal and verbal feedback” (Gabel and Robb, 2017, p. 129). The findings reveal an interplay between certain senses, imagination, and active creation with the art form (Barak and Stebbins, 2017).

*Creativity* includes being open to new and more adaptive possibilities (Orkibi, 2021), through active play-like exploration, self-expression, testing and trying out new ways of being. This finding is important within the integrated CATs approaches, because the shift from one art medium to another promotes different perspectives, and opens up different artistic pathways to therapeutic goals (Ram-Vlasov and Orkibi, 2021). In their summary of CATs definitions, Karkou and Sanderson (2006) highlighted creativity as one of several key therapeutic agents of change.

*Artistic pleasure* (Rylatt, 2012) is another JF identified in the data. The unique engagement in the process of CATs may afford participants joyful and pleasant experiences that can instill hope and optimism (Azoulay and Orkibi, 2015; Orkibi, 2019). Playfulness is an important component of this process, which may be enhanced by movement and dance, spontaneous improvisation and role-play, the use of art materials, and/or playing instruments in a safe and nonjudgmental space. The experience of beauty is also included in this domain as a potential source of pleasure (Mannheim et al., 2013; Koch, 2017).

*Modulating time and space* reflects being able to work in the here-and-now as well as at times in the there-and-then, thus enabling clients to transcend the limitations of time and place within a creative space that facilitates the potential for change and growth using imagination (Moreno, 1965; Winnicott, 1980; Pendzik, 2006). Some CATs scholars connect this with the state of flow (Baker et al., 2015; Abbing et al., 2018), while others refer to the ability of the arts and arts-making to act as a temporary distraction from illness (Flanagan, 2004; Porter et al., 2017), which possibly has to do with the expression of the multiple pathways in which the mind-body connection occurs in CATs contexts.

*Non-verbal expression* allows clients to externalize all internal experiences, especially with regards to challenging features, and to articulate them in words or by other visual, audio or kinesthetic means (Kaimal et al., 2017; Smith et al., 2017; Lauffenberger, 2020).

Overall, the results provided insights into the role and function of the specific and joint factors in CATs such as embodiment, concretization, symbolism, and metaphors. The results also identified mixed therapeutic factors (Koch, 2014; Wiedenhofer and Koch, 2017; De Witte et al., 2020b), involving the reinforcement of common psychotherapy factors. However, these instances are also connected to intra- and inter-artistic factors within and between each discipline. This underscores the importance of connecting these mixed CAT therapeutic factors and how they reflect more recent CAT change models such as Jones (2007, 2021) and Koch (2017).

### Specific Factors of Each CATs Discipline

#### Art Therapy

The findings showed that specific factors are related to artwork and art-making such as promoting positive therapeutic change by seeing one's own emotions through the artwork, eliciting a “visual narrative of life” and “portraying the feelings of past/future” (Haeyen et al., 2015; Hilbuch et al., 2016; Bosgraaf et al., 2020). The artwork, in this way, becomes a tangible and concrete agent that facilitates perspective taking, motivates self-reflection and self-awareness, and further promotes understanding during the therapeutic process (Abbing et al., 2018; Bosgraaf et al., 2020). This finding aligns with the recent Adaptive Response Theory art therapy framework, which situates art-making as well as the art product as distinct elements that enable clients to shift from maladaptive to adaptive responses through the interpersonal and intrapersonal understanding that occurs in the session (Kaimal, 2019).

Another important domain of specific factors in art therapy is interaction and engagement through the physicality of the various art materials. Discovering materials and possibilities with art media (Haeyen et al., 2015) have been associated with a sense of mastery, embodied imagination, and artistic agency. The tactile quality of the art media (Abbing et al., 2018) and the use of specific art materials and techniques (Bosgraaf et al., 2020) facilitates exploration and creative engagement during the art therapy process. This is consistent with the theoretical model of engagement with the art media at various kinesthetic/sensory, perceptual/affective, and cognitive/symbolic levels that are all linked at the creative level (Lusebrink et al., 2013). However, the specific mechanisms by which the different dimensions of the artistic medium facilitate various levels of engagement deserve further theorization and empirical investigation.

#### Dance Movement Therapy

The *non-verbal nature* of DMT is considered an important therapeutic factor (Mannheim et al., 2013; Shim, 2015; Chyle et al., 2020). As the definition of the discipline suggests (Karkou and Sanderson, 2006), attention to the body and movement allows for body-mind connections and for holistic attention to therapeutic change. Similarly, *embodiment*, in the form of *experiencing the body* (Chyle et al., 2020) and *body awareness* (Mannheim et al., 2013; Shim, 2015), is an important factor of change in the DMT studies included here. This finding echoes theoretical discussions on embodiment in the discipline (Koch and Fuchs, 2011; Fuchs and Koch, 2014; Payne et al., 2019) as well as applications of these ideas to clinical practice (Sandel et al., 1993; Pallaro, 2007) and research (Meekums et al., 2015; Pylvänäinen et al., 2015; Pylvänäinen, 2018; Karkou et al., 2019). Mary Whitehouse (Pallaro, 2007) for example, one of the early pioneers of DMT in the USA, and a Jungian analyst, highlighted the value of heightening sensorial messages as a way to provide information in the here-and-now, but also as a way to allow one to delve into the depth of one's inner world and past history. Similarly, Ellis' (2001) discussion of movement metaphors refers directly to revealing *unconscious processes*, and Chyle et al's (2020) references to bodily sensations are regarded as supporting *insight*. Both findings echo influences of Jungian thinking and depth psychology in DMT practice and support arguments that the discipline is a form of creative psychotherapy where movement metaphors play a central role (e.g., Meekums, 2002).

Influences from movement analysis systems and movement-based practices such as Laban (McCaw, 2012) and Kestenberg Amighi et al. (2018) can also be identified in the specific DMT factors. Chyle et al. (2020), for example, noted the development of *artistic skills* including connecting movement with language and learning certain movement sequences, while Koch et al. (2007) explored the role of the jumping rhythm for developing *artistic* and *general agency* as described by Kestenberg Amighi et al. (2018).

Finally, mirroring (Shuper-Engelhard et al., 2019), movement interactions and movement synchronicity (Chyle et al., 2020) all emerged as important therapeutic factors. The development of the *therapeutic alliance, dialogue* and *synchrony* are part of the fabric of DMT practice, and were strongly advocated by Marian Chace and her students and explicitly flagged up by Schmais (1985) in one of the first texts on therapeutic factors written in the discipline. Schmais (1985), in this seminal text, talked about healing processes in DMT, translating Yalom's therapeutic factors in verbal psychotherapy groups into DMT group practice. The assumption that these are important and unique ingredients of DMT that are responsible for therapeutic change remains largely the same. Conversely, the contemporary literature translates practice-based theorization into empirical investigations, as evidenced by the DMT publications included in this review.

#### Drama Therapy and Psychodrama

Overall, we identified more PD than DT specific factors. Beyond the fact that there were more PD than DT studies included in this review, PD is more structured and unified than DT in terms of both theory and practice (Kedem-Tahar and Kellermann, 1996). Therefore, the therapeutic factors in PD are relatively well-defined and are often quantitatively measured through observational ratings or self-reports. As such, they lend themselves more readily to consistent operationalization and empirical investigation. For instance, *doubling* and *role reversal* are well-defined PD techniques, while *encounter, role-reconstruction*, and *catharsis* are well-defined key factors in the PD theory of change (Moreno, 1972/1994; Blatner, 2000; Orkibi, 2019). PD therapeutic factors that call for further empirical investigation include spontaneity and creativity, concretization, act hunger, act fulfillment, and action insight, to name a few.

In contrast, the DT therapeutic factors of dramatic projection and embodiment, which were identified in qualitative studies, need further operationalization before they can be quantitatively measured in future change process or process-outcome studies. For example, Jones (2007) suggested the following eight core processes that apply to all DT approaches and reflect in-session drama and theater therapeutic processes: dramatic projection, drama therapeutic empathy and distancing, role-playing and personification, interactive audience and witnessing, embodiment, playing, the life-drama connection, and transformation (pp. 99–129). Finally, further operationalization of Landy's (1997) concept of aesthetic distance may provide a discipline-specific conceptualization for clients' levels of emotional involvement in a DT session that may be associated with a range of meaningful psychological outcomes such as emotion regulation.

#### Music Therapy

The findings show that specific factors related to the *musical dialogue*, such as “shared musical experiences” or “musical interactions,” are very often associated with positive therapeutic change. This suggests that the therapeutic relationship itself is formed *in* the music. By offering a solid musical frame, any musical expression produced by the client can be musically encouraged and responded to in a musical dialogue (Nordoff and Robbins, 1966; Aigen, 2005; MacDonald et al., 2013). The 64 musical improvisation techniques developed by Bruscia (1987) based on using the unique qualities of music to establish/influence the musical dialogue, are still the basis of music therapy training all over the world. However, since musical dialogue contains so many different components and working processes, both inside and outside the music, it is strongly recommended to conduct further research into related therapeutic factors, for example through micro-analysis of a short segment of a session (Lee, 2000; Wosch and Wigram, 2007).

Another important domain of specific factors in music therapy associated with a wide range of positive outcomes (e.g., reduction of stress, arousal, anxiety), concerns the *structuring nature of music*, which were referred to in the studies as “repetitive rhythms,” “tempo of the music,” and “musical simplicity.” Neurological evidence shows that music with a slow and steady rhythm can provide relaxation and calm by altering autonomic body rhythms such as heart rate (e.g., Thaut et al., 1999, 2015; Bernardi et al., 2006; Thaut and Hoemberg, 2014; Koelsch, 2015). This is in line with two recent meta-analyses which reported positive correlations between a slow, steady music tempo and stress/anxiety reduction (De Witte et al., 2020a,b). However, most of these studies used pre-recorded music and music tempo was usually not measured during a music therapy session using improvised live music. Therefore, we recommend further investigation of music tempo as a specific therapeutic factor within active music therapy interventions, as related to psychophysiological and psychological outcomes.

### Implications for Clinical Practice and Education

While there is literature on CATs education in each discipline, there is much less on joint CATs pedagogy. The 19 domains that emerged from the CATs literature can contribute to closing this gap. These 19 domains build upon the state of the art in terms of the role and function of the arts and arts-based learning in CATs (Landy et al., 2005, 2012; Gerber, 2006; Deaver, 2012; Young, 2012; Hahna, 2013; Knight and Matney, 2014; Butler, 2015, 2017a,b; Gaines et al., 2015; Demaine, 2016). There has also been a small although growing dialogue on CATs online and distance learning pedagogy where our results can also inform teaching practices. This is particularly true regarding closer connections between joint CATs factors and their impacts on clinical skills such as intentionality, presence, and evaluation in distance-learning contexts (Vega and Keith, 2012; LaGasse and Hickle, 2015; Beardall et al., 2016; Blanc, 2018; Sajnani et al., 2019, 2020; Pilgrim et al., 2020). Some studies can contribute directly to pedagogy in areas such as the student experience and student development (Orkibi et al., 2021), curricular and program development (Moon, 2003), field training approaches and models (Fish, 2008; Landy et al., 2012; Orkibi, 2012) and program evaluation and assessment protocols (Julliard et al., 2000; Cruz, 2013). For example, CATs educators can associate the change process to certain CATs models, such as the five domains of change (Koch, 2017) and the Expressive Therapies Continuum (Lusebrink et al., 2013). This way of conceptualizing CATs education may provide new teaching and learning perspectives where CATs education can occur within shared CATs frameworks.

The 19 domains can also constitute a common language for clinical training. This includes connections to the local and global CATs community and its cross-cultural implications for teaching and learning. Current areas of global concern are the field's cultural responses to health issues and health crises (Harvey et al., 2020), and the psycho-physiological impacts of illness (Czamanski-Cohen et al., 2020). Understanding the impact and effect of common CATs and specific factors in CATs can provide advanced training tools for educators to tailor interventions in specific cultural contexts. Further advances in inclusive clinical CATs program site development for schools (Hannigan et al., 2019) is also a current concern. Such considerations for programs reimagine the role and function of other joint CATs factors such as creativity and emotions in curriculum planning, community mental health, and student wellness (Quinlan et al., 2016; Hannigan et al., 2019). Finally, the results capture the unique characteristics of CATs and contribute toward enhancing ongoing dialogues on pedagogical frameworks in education and leadership in CAT (Kaimal et al., 2017).

### Implications for Future Research

#### Terminology and Theory

This scoping review underscores the complexity of studying therapeutic factors. Overall, there is a need for a more unified vocabulary and a greater specificity in conceptualizing and operationalizing therapeutic factors in CATs. One necessary starting point is a shared terminology (i.e., Table 2) used in an accurate, systematic, and consistent way by the CATs research community. Shared definitions help recognize and minimize redundancy, confusion, and contraindications. For example, in some studies referring to a measured outcome variable (e.g., enhanced self-concept, emotion regulation, empowerment, stress) as a therapeutic factor is questionable given the fact that such variables are more often than not defined in the psychology and psychotherapy literature as outcomes rather than therapeutic factors. Nevertheless, a compelling theoretical argument for conceptualizing such outcome variables as therapeutic factors was also often found to be lacking.

In line with Kazdin (2007, 2009), it is crucial to use theory as a guide to explain how a therapeutic factor operates across CATs and in a given discipline. A theory can be studied bottom-up (by exploratory qualitative or quantitative data) and/or top-down (by an existing theory of change). For example, Blatner (1992) described the underlying therapeutic principles of all CATs and Johnson (1998) introduced a psychodynamic model that explains CATs' therapeutic action. More recently, Koch (2017) suggested the framework of embodied aesthetics as a model for specific mechanisms in CATs. There are also discipline-specific models of therapeutic factors; for example, in drama therapy (Jones, 2007), psychodrama (Kushnir and Orkibi, 2021), art therapy (Czamanski-Cohen and Weihs, 2016; Kaimal, 2019), dance movement therapy (Koch and Fischman, 2011; Imus, 2021), and music therapy (Baker and Roth, 2004; Grocke, 2019).

#### Methodological Implications and Recommendations

**Figure 4:**
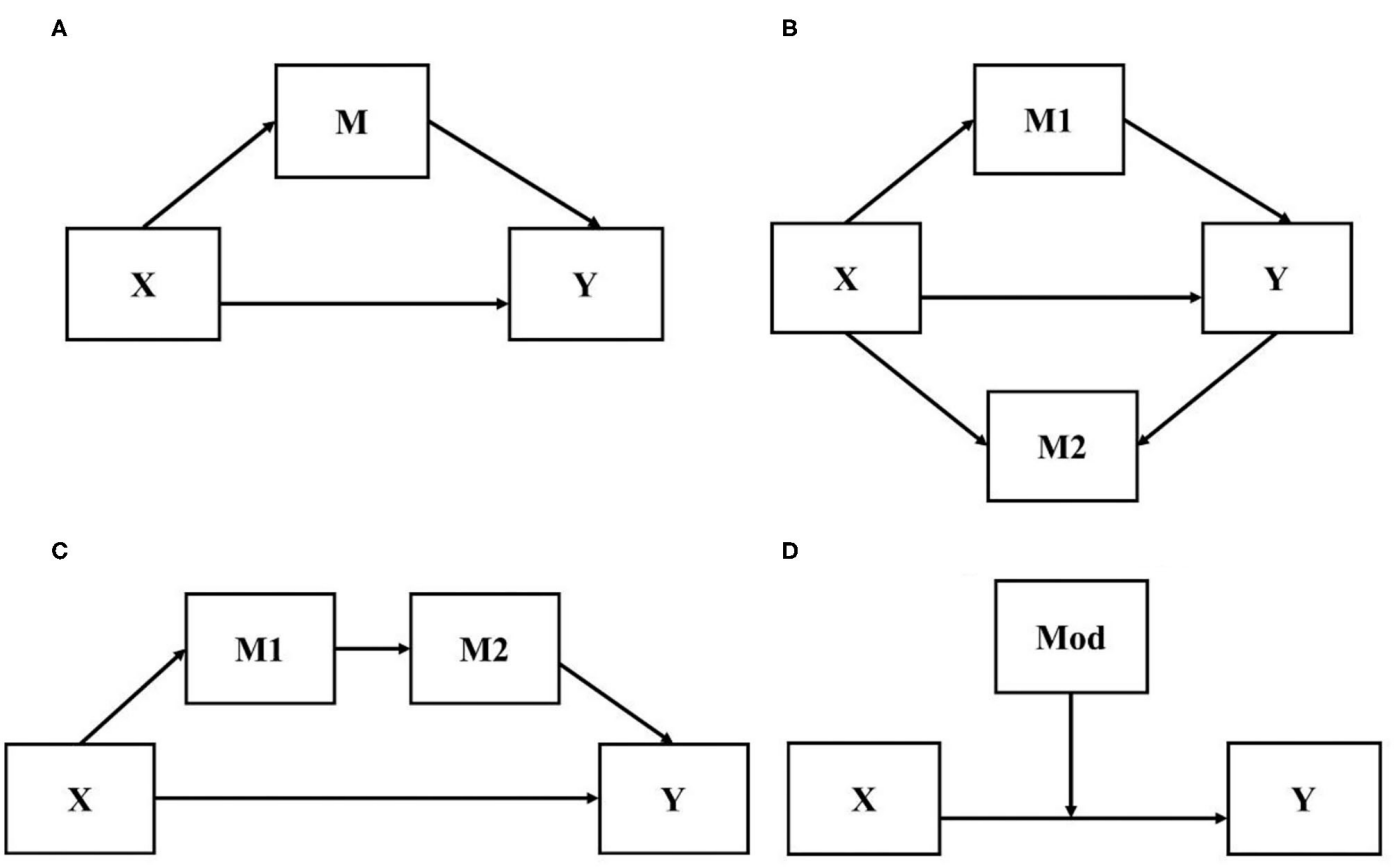
Types of Meditation and Moderation Models. **(A)** Simple mediation. **(B)** Parallel mediation. **(C)**. Sequential mediation. **(D)** Moderation. X = independent variable (mostly the form of therapy or intervention), M = Mediator (the change factor operationalized into a mediator), Y = dependent variable/outcome (e.g., well-being), Mod = Moderator (for additional mediation and moderation models see Hayes, 2013).

To examine how change occurs, psychotherapy researchers often look at *mediators*, but this line of studies is generally lacking in CATs studies and therefore calls for further discussion here. A mediator (M) is an intervening variable that is theorized to account for the causal statistical relationship between two variables, such that X causes M which in turn causes Y (see Figure 4A). In psychotherapy research, a mediator is situated between the intervention (independent variable, X) and the outcome (dependent variable, Y) (Kazdin, 2009). For example, a psychodrama intervention (X) may increase perspective taking (Y) through role reversal (M). It is possible to examine a more complex theory of change that has parallel mediators or a chain of sequential mediators in a single statistical model, as illustrated in Figures 4B,C (see also Hayes, 2013). Thus, we propose reserving the term mediator or mediating variable for the statistical relationship between variables, in the context of statistical mediation analysis.

However, “mediation does not equal mechanism” (Nock, 2007, p. 5S), because isolating a mediator does not itself explain how treatment leads to change. Kazdin (2009) noted that while the study of mediation is the first step in understanding how therapy works, “mechanism refers to a greater level of specificity than a mediator and reflects the steps or processes through which therapy (or some independent variable) actually unfolds and produces the change” (p. 419). Proposing a mechanism of change is first and foremost a theoretical task that should reflect the theory of change posited by the researcher. This includes a causal sequence of “events” (or mediators in quantitative mediation analysis) that reflect how the change occurred. To illustrate, a strong client-therapist alliance (a common change factor serving here as an independent variable), may lead to more dramatic engagement in drama (a specific change factor, M1) that in turn leads to more action insight (another specific change factor, M2), which enhances self-understanding (a common change factor, M3), and consequently to behavioral change (the dependent variable). This example illustrates a causal process (i.e., sequential chain of variables) reflecting one possible mechanism of change in psychodrama that involves both common and specific factors. Note that the independent variable can also be the intervention (e.g., psychodrama vs. CBT) such that the treatment variable precedes the alliance. Mediators should be clearly articulated in the theoretical model upon which the treatment is based in order to establish “treatment differentiation” that clarifies the essential factors by which a given intervention differs from a comparison or control group (Ang et al., 2018). Creative self-efficacy is a particularly relevant construct that offers promising avenues for future CATs research on mechanisms, because it has been recently associated with creative adaptability and well-being indicators (Orkibi, 2021).

A *moderator* is a construct external to the treatment that influences the direction or magnitude of the relationship between the treatment and outcome (see Figure 4D). Hypothesizing moderators can clarify when or for whom therapy leads to change, because moderators are often client or therapist characteristics (e.g., gender, ethnicity, years of practice, temperament) or the treatment delivery format (e.g., individual vs. group; in-person vs. online) and dose (e.g., once vs. twice a week) (Kazdin, 2009). It is noteworthy that in some cases “a given variable may function as either a moderator or a mediator, depending on the theory being tested” (Frazier et al., 2004, p. 116). More complex statistical analyses can examine a *moderated mediation* model in which “the effect of X on Y through M is moderated by or conditioned on one or more variables” (Hayes, 2013, p. xi). In other words, the mediation “operates to varying degrees (or not at all) for certain people or in certain contexts” (Hayes, 2013, p. 432). In a *mediated moderation* model, “the interaction between two variables affects a mediator, which then affects a dependent variable” (Morgan-Lopez and MacKinnon, 2006, p. 77). However, it has been claimed that the latter model “rarely has a meaningful substantive interpretation” and thus should be avoided (see Hayes, 2013).

Note that while qualitative research does not involve statistical analysis of possible causal relationships between variables, empirical qualitative research draws on participants' and/or the therapists' self-reports of their treatment experiences to describe possible therapeutic factors, or more detailed mechanisms of change, and vice-versa. Relatedly, labeling the therapeutic factors identified in a qualitative study as mediators should be avoided, since this term is conventionally used in the context of quantitative statistical analysis.

We call on CATs researchers to include two or more measures of candidate mediators in quantitative treatment studies in order to identify which mediator makes a greater contribution to the outcome. Mediators may be related to the therapist, the client, the interaction, and/or the group. However, to demonstrate a causal timeline (i.e., temporal sequence) it is important to assess both the mediator and the outcome (preferably in multiple occasions across the treatment), “to ensure the mediator has, in fact, changed before the outcome” (Kazdin, 2009, p. 424). Both control studies (in a laboratory) and naturalistic studies (in a real-world setting) are valuable to identify mediators and therapeutic factors. In controlled studies, the direct experimental manipulation of a proposed mediator can provide evidence that the change in the outcome is a function of the levels of the manipulated mediator (e.g., a group with low vs. a group with high dramatic engagement). Naturalistic studies can be useful as well for generating and testing hypotheses about therapeutic factors in treatments delivered in the field (Kazdin, 2009). The conditions under which a therapeutic factor impacts a given outcome (i.e., when or for whom) can be assessed with a conditional meditation analysis (i.e., “moderated mediation”) with the mediator being specified, for example, as a dose (e.g., treatment length), different client characteristics (e.g., age, gender), and varying settings (e.g., individual, group).

Moreover, the CATs field can progress through research that pinpoints therapeutic factors in the artistic and creative process, thus pointing to CATs as treatment methods in their own right. To this end, we urge researchers to develop methodologically rigorous measures that capture CATs' unique therapeutic factors, including those designed to determine the embodied and non-verbal expressive and creative processes that can be assessed by means of triangulation to strengthen internal validity (e.g., self-reported, therapist reported, and observational data). These measures should be disseminated to the CATs research community, and in particular to faculty members who are active researchers in research-oriented CATs programs, who may help establish their validity, reliability, and replicability across settings and populations.

Finally, the development and testing of CAT micro-interventions responds to the call for more research on therapeutic factors. Micro-interventions can be regarded as a short part of a session in which the therapist uses specific therapeutic techniques or steps to work on the client's specific goals (De Witte et al., submitted), which is in line with the growing recognition that one-size-fits-all approaches to intervention may be suboptimal for the client and healthcare system alike (Rush et al., 2004; Gauthier et al., 2017). Developing CAT micro-interventions may be a suitable way to uncover a therapist's implicit and practice-based knowledge, which may stimulate the transferability of valuable clinical practices and provide more insights into which factors lead to change. Micro-intervention testing also allows researchers to conduct micro-analyses on a very short segment of the therapeutic process and is therefore highly suitable for pinpointing elements of the intervention that cannot be examined when testing over a larger period of time. Ultimately, such knowledge exchange will promote the consistency and replication of results across independent studies, thus enhancing possibilities to draw substantiated inferences about a given therapeutic factor (Kazdin, 2009).

### Study Limitations

There are several limitations to this study. Because we included studies where therapeutic factors were considered as a framework, as well as studies explicitly addressing therapeutic factors in their research questions (i.e., they actually examined therapeutic factors), the data across studies are diverse. Our searches may have failed to identify studies where the researchers did not include keywords or terms that indicate they researched therapeutic factors. In addition, not all CATs disciplines were included (i.e., poetry-/biblio-therapy), and only data-driven published studies met the criteria. Articles on theoretical descriptions of therapeutic factors without empirical testing were also excluded. Thus, it is not impossible that some outcome studies that did not use the terms related to therapeutic factors (e.g., Table 2) may not have been identified. Furthermore, the diversity of orientations across the CATs presented challenges when attempting to identify mediators, moderators, and outcomes and how to label them as factors and frameworks which ranged from aesthetics, psychotherapeutic, to biomarkers and neuroscience. As a result, a broad swath of joint CATs factors and specific factors with varying degrees of specificity were identified. To keep the review manageable, certain details on techniques and interventions and their relationship to mechanism activation were lost in the synthesis process. For example, Abbing et al. (2018) described common factors of cognitive regulation with a further distillation to AT specific factors of “reflections upon art.” Our review did not describe these in detail, nor the therapy orientation of these AT researchers.

This scoping review constructed a comprehensive list of therapeutic factors and leaves to future research endeavors the in-depth description of how, why and under what conditions these therapeutic factors are activated. Readers are encouraged to read the tables in Supplementary Materials and each study cited to obtain a more detailed descriptions of each therapeutic factor and/or mechanism of change. The need for synthesis at this level highlights the importance for each discipline to undertake more in-depth analyses of the therapeutic factors described in the studies cited here. An important point to consider is that mechanisms are constructs of clinical practice. On the conceptual level, there is always surplus meaning related to each therapeutic factor, even the physiological ones. On the methodological level, quantitative mechanism research is limited to a linear cause and effect logic, as is outcome research, and in studies within CATs' reach it is often only possible to test one factor at a time. This means that parallel and sequential factors are hard to identify, and more complex systemic and circular relations, as assumed in some CAT mechanism models, cannot be represented by existing mechanism models (Kazdin, 2007; Hayes, 2013). While mechanism research is starting to look at more complexity in therapeutic processes, it is important to acknowledge that its quantitative version is limited in similar ways as is outcome research.

Finally, our research team was composed of members from the US, UK, the Netherlands, Germany, Israel, Hong Kong China, and Australia, so that the articles reviewed and included were limited to those written in English. A body of research published in other languages may have been omitted due to language barriers, particularly from the Nordic countries that are active in CATs research, and countries from South America, Africa, Asia, and the Middle East, where some researchers do not publish in English. CATs practices may differ as a function of cultural differences in the use of the creative and performing arts in those countries, so there is a gap in identified therapeutic factors that may be culturally specific to those groups. Any future systematic review would benefit from an even more global review team, or a specialized review, to ensure a multicultural outlook.

## Concluding Remarks

Change process research is crucial to the advancement of CATs. It can help to: (a) identify specific therapeutic factors that can account for the ways in which therapeutic change occurs, (b) improve the effectiveness of interventions by focusing on these therapeutic factors, (c) refine a theory of change that provides an underpinning rationale and structure for the treatment, and (d) develop more effective training and supervision on effective therapeutic factors that are supported by evidence (Hardy and Llewelyn, 2015). This review brought us closer to answering the question of which possible mechanisms of change connect CATs interventions with outcomes. We were able to strengthen the connections between our theoretical models in CATs and specific CATs therapeutic factors. In so doing, the level of specificity improved, which supports the foundations for change process research in the CATs.

The results point to avenues for future studies on mechanisms of change that will lead to a better understanding of CATs-specific factors. This study revealed two key areas of consideration to move the field forward: the joint factors in CATs and the specific factors for each discipline in CATs. Shaping a change process research program in the CATs will be a challenge due to the level of complexity and diversity in these two key areas. Kazdin (2009) discussed the challenges this poses with a particular focus on its impact on the quality of care. He suggested that “the complexities are critically important to understand, because the best patient care will come from ensuring that the optimal variation of treatment is provided” (p. 426). This is a crucial concern regarding the non-linear creative processes in CATs context and the need to allow for this in research design and methods. We were able to identify levels of general consistency with CATs factors and can infer a certain level of involvement of these components as potential mediators and/or mechanisms. However, this major finding also points to the lack of specificity and focus on change processes in study design and methodology. It is therefore critical to begin a change process research program with a strategy that shapes pathways to locate and illuminate mechanisms and mediators in CATs. As the first step, however, CATs researchers need to conceptualize their theory of change, building on existing models (e.g., Johnson, 1998; Koch, 2017; Jones, 2021), formulate questions that address treatment change, and elaborate on the possible mechanisms that may account for this change, and on how they operate.

In summary, this scoping review provides an initial framework featuring empirical clusters of relevant therapeutic factors that can encourage researchers to begin to develop process-outcome and change process research programs in the CATs. Our framework can serve as a foundation to build pathways toward a greater understanding of joint and specific factors in the CATs, which are attributed to the therapist, the client, the artwork, and the interaction between them in CATs-specific contexts. This will ultimately advance CATs research in moving from the study of therapeutic factors to a more complex examination of mechanisms of therapeutic change.

## Data Availability Statement

The original contributions generated for the study are included in the article/Supplementary Materials, further inquiries can be directed to the corresponding authors.

## Author Contributions

All authors listed have made a substantial, direct and intellectual contribution to the work, and approved it for publication.

## Conflict of Interest

The authors declare that the research was conducted in the absence of any commercial or financial relationships that could be construed as a potential conflict of interest.

## Footnotes

^1^For more information see: https://rayyan.qcri.org.

## References

[B1] Abbing A. Ponstein A. van Hooren S. de Sonneville L. Swaab H. Baars E. (2018). The effectiveness of art therapy for anxiety in adults: a systematic review of randomised and non-randomised controlled trials. PLoS ONE 13:e0208716. 10.1371/journal.pone.0208716 30557381 PMC6296656

[B2] Ahonen-Eerikäinen H. Rippin K. Sibille N. Rhea K. (2007). “Not bad for an old 85-year-old!” - the qualitative analysis of the role of music, therapeutic benefits and group therapeutic factors of the St. Joseph's Alzheimer's adult day program music therapy group. Can. J. Music Ther. 13, 37–62.

[B3] Aigen K. (2005). Music-Centered Music Therapy. New Braunfels: Barcelona Publishers.

[B4] Ang K. Hepgul N. Gao W. Higginson I. J. (2018). Strategies used in improving and assessing the level of reporting of implementation fidelity in randomised controlled trials of palliative care complex interventions: a systematic review. Palliat. Med. 32, 500–516. 10.1177/0269216317717369 28691583

[B5] Ansdell G. Davidson J. Magee W. Meehan J. Proctor S. (2010). From 'this f^***^ing life' to 'that's better'. in four minutes: an interdisciplinary study of music therapy's 'present moments' and their potential for affect modulation. Nordic J. Music Ther. 19, 3–28. 10.1080/08098130903407774

[B6] Arksey H. O'Malley L. (2005). Scoping studies: towards a methodological framework. Int. J. Soc. Res. Methodol. 8, 19–32. 10.1080/1364557032000119616

[B7] Armstrong C. R. Rozenberg M. Powell M. A. Honce J. Bronstein L. Gingras G.. (2016). A step toward empirical evidence: operationalizing and uncovering drama therapy change processes. Arts Psychother. 49, 27–33. 10.1016/j.aip.2016.05.007

[B8] Arnheim R. (1980). Art as therapy. Arts Psychother. 7, 247–251. 10.1016/0197-4556(80)90001-5

[B9] Azoulay B. Orkibi H. (2015). The four-phase CBN Psychodrama model: a manualized approach for practice and research. Arts Psychother. 42, 10–18. 10.1016/j.aip.2014.12.012

[B10] Backett K. C. Davison C. (1995). Lifecourse and lifestyle: the social and cultural location of health behaviours. Soc. Sci. Med. 40, 629–638. 10.1016/0277-9536(95)80007-7 7747198

[B11] Baker F. A Rickard N Tamplin J Roddy C. (2015). Flow and meaningfulness as mechanisms of change in self-concept and well-being following a songwriting intervention for people in the early phase of neurorehabilitation. Front. Hum. Neurosci. 9:299. 10.3389/fnhum.2015.00299 26082702 PMC4443737

[B12] Baker F. A. Roth E. (2004). Neuroplasticity and recovery: training models and compensatory strategies in music therapy. Nordic J. Music Ther. 13, 20–32. 10.1080/08098130409478095

[B13] Barak A. Stebbins A. (2017). Imaginary dialogues: witnessing in prison-based creative arts therapies. Arts Psychother. 56, 53–60. 10.1016/j.aip.2017.07.003

[B14] Beardall N. Blanc V. Cardillo N. J. Karman S. Wiles J. (2016). Creating the online body: educating dance/movement therapists using a hybrid low-residency model. Am. J. Dance Ther. 38, 407–428. 10.1007/s10465-016-9228-y

[B15] Bernardi L. Porta C. Sleight P. (2006). Cardiovascular, cerebrovascular, and respiratory changes induced by different types of music in musicians and nonmusicians: the importance of silence. Heart 92, 445–452. 10.1136/hrt.2005.064600 16199412 PMC1860846

[B16] Bibb L. McFerran K. S. (2018). Musical recovery: the role of group singing in regaining healthy relationships with music to promote mental health recovery. Nordic J. Music 27, 235–251. 10.1080/08098131.2018.1432676

[B17] Blanc V. (2018). The experience of embodied presence for the hybrid dance/movement therapy student. Internet Higher Educ. 38, 47–54. 10.1016/j.iheduc.2018.05.001

[B18] Blatner A. (1992). Theoretical principles underlying creative arts therapies. Arts Psychother. 18, 405–409. 10.1016/0197-4556(91)90052-C

[B19] Blatner A. (2000). Foundations of Psychodrama: History, Theory, and Practice, 4th Edn. New York, NY: Springer.

[B20] Bonde L. O. (2005). Finding a New Place.” metaphor and narrative in one cancer survivor's BMGIM therapy. Nordic J. Music Ther. 14, 137–154. 10.1080/08098130509478135

[B21] Bosgraaf L. Spreen M. Pattiselanno K. Hooren S. V. (2020). Art therapy for psychosocial problems in children and adolescents: a systematic narrative review on art therapeutic means and forms of expression, therapist behavior, and supposed mechanisms of change. Front. Psychol. 11:2389. 10.3389/fpsyg.2020.584685 33132993 PMC7578380

[B22] Bruscia K. E. (1987). Improvisational Models of Music Therapy. Springfield, IL: Charles C. Thomas.

[B23] Bucută M. D. Dima G. Testoni I. (2018). “When you thought that there is no one and nothing”: the value of psychodrama in working with abused women. Front. Psychol. 9:1518. 10.3389/fpsyg.2018.01518 30190692 PMC6115514

[B24] Butler J. D. (2015). “Playing with reflection in drama therapy education,” in Playing in a House of Mirrors, eds E. Vettraino, and W. Linds (Rotterdam: Sense Publishers), 109–122.

[B25] Butler J. D. (2017a). Re-examining Landy's four-part model of drama therapy education. Drama Ther. Rev. 3, 75–86. 10.1386/dtr.3.1.75_1

[B26] Butler J. D. (2017b). The complex intersection of education and therapy in the drama therapy classroom. Arts Psychother. 53, 28–35. 10.1016/j.aip.2017.01.010

[B27] Carr C. d'Ardenne P. Sloboda A. Scott C. Wang D. Priebe S. (2012). Group music therapy for patients with persistent post-traumatic stress disorder - an exploratory randomized controlled trial with mixed methods evaluation. Psychol. Psychother. Theory Res. Prac. 85, 179–202. 10.1111/j.2044-8341.2011.02026.x 22903909

[B28] Carroll L. (1971/1865). Alice in Wonderland. New York, NY: Norton.

[B29] Cassidy S. Gumley A. Turnbull S. (2017). Safety, play, enablement, and active involvement: themes from a grounded theory study of practitioner and client experiences of change processes in Dramatherapy. Arts Psychother. 55, 174–185. 10.1016/j.aip.2017.05.007

[B30] Cassidy S. Turnbull S. Gumley A. (2014). Exploring core processes facilitating therapeutic change in dramatherapy: a grounded theory analysis of published case studies. Arts Psychother. 41, 353–365. 10.1016/j.aip.2014.07.003

[B31] Chiang M. Reid-Varley W. B. Fan X. (2019). Creative art therapy for mental illness. Psychiatry Res. 275, 129–136. 10.1016/j.psychres.2019.03.025 30901671

[B32] Chyle F. Boehm K. Imus S. Ostermann T. (2020). A reconstructive hermeneutic analysis: the distinctive role of body- and movement-based interventions with male offenders. Body Movement Dance Psychother. 15, 106–123. 10.1080/17432979.2020.1748902

[B33] Clift S. Camic P. M. (2016). “Introduction to the field of creative arts, wellbeing, and health: achievements and current challenges,” in Oxford Textbook of Creative Arts, Health and Wellbeing. International Perspectives on Practice, Policy, and Research, eds S. Clift and P. M. Camic (Oxford: Oxford University Press), 4–9.

[B34] Crits-Christoph P. Connolly Gibbons M. B. Mukherjee D. (2013). “Psychotherapy process-outcome research,” in Bergin and Garfield's Handbook of Psychotherapy and Behavior Change, 6th Edn., ed M. J. Lambert (Hoboken, NJ: John Wiley and Sons), 298–340.

[B35] Cruz R. F. (2013). Feders' the Art amd Science of Evaluation in the Arts Therapies: How Do You Know What's Working. Springfield, IL: Charles C. Thomas.

[B36] Cuijpers P. Reijnders M. Huibers M. J. H. (2019). The role of common factors in psychotherapy outcomes. Annu. Rev. Clin. Psychol. 15, 207–231. 10.1146/annurev-clinpsy-050718-095424 30550721

[B37] Cuijpers P. Van Straten A. Warmerdam L. (2007). Behavioral activation treatments of depression: a meta-analysis. Clin. Psychol. Rev. 27, 318–326. 10.1016/j.cpr.2006.11.001 17184887

[B38] Czamanski-Cohen J. Weihs K. L. (2016). The bodymind model: a platform for studying the mechanisms of change induced by art therapy. Arts Psychother. 51, 63–71. 10.1016/j.aip.2016.08.006 27777492 PMC5074079

[B39] Czamanski-Cohen J. Wiley J. Weihs K. (2020). Protocol for the REPAT study: role of emotional processing in art therapy for breast cancer palliative care patients. BMJ Open 10:e037521. 10.1136/bmjopen-2020-037521 33444178 PMC7678396

[B40] Czamanski-Cohen J. Wiley J. F. Sela N. Caspi O. Weihs K. (2019). The role of emotional processing in art therapy (REPAT) for breast cancer patients. J. Psychosoc. Oncol. 37, 586–598. 10.1080/07347332.2019.1590491 30929590

[B41] Dallos R. Vetere A. (2005). Researching Psychotherapy and Counselling. Maidenhead: Open University Press.

[B42] Dalton T. A. Krout R. E. (2015). The grief song-writing process with bereaved adolescents: an integrated grief model and music therapy protocol. Music Ther. Perspect. 24, 94–107. 10.1093/mtp/24.2.94

[B43] De Felice G. Giuliani A. Halfon S. Andreassi S. Paoloni G. Orsucci F. F. (2019). The misleading dodo bird verdict. How much of the outcome variance is explained by common and specific factors? New Ideas Psychol. 54, 50–55. 10.1016/j.newideapsych.2019.01.006

[B44] De Witte M. da Silva Pinho A. Stams G-J. Moonen X. Bos E. R. A. van Hooren S. (2020b). Music therapy for stress reduction: a systematic review and meta-analysis. Health Psychol. Rev. 31, 1–26. 10.1080/17437199.2020.1846580 33176590

[B45] De Witte M. Lindelauf E. Moonen X. Stams G. J. van Hooren S. (2020c). Music therapy interventions for stress reduction in Adults with Mild Intellectual Disabilities: perspectives from clinical practice. Front. Psychol. 11:572549. 10.3389/fpsyg.2020.572549 33362637 PMC7759728

[B46] De Witte M. Spruit A. van Hooren S. Moonen X. Stams G-J. (2020a). Effects of music interventions on stress - related outcomes: a systematic review and two meta analyses. Health Psychol. Rev. 14, 294–324. 10.1080/17437199.2019.1627897 31167611

[B47] Deaver S. P. (2012). Art-based learning strategies in art therapy graduate education. Art Ther. 29, 158–165. 10.1080/07421656.2012.730029

[B48] Deboys R. Holttum S. Wright K. (2017). Processes of change in school-based art therapy with children: a systematic qualitative study. Int. J. Art Ther. 22, 118–131. 10.1080/17454832.2016.1262882

[B49] Demaine K. (2016). A reunion of east and west: reflections on the roots of creative arts therapy and traditional chinese medicine. Creative Arts in Educ. Ther. 2, 29–39. 10.15534/CAET/2016/1/25

[B50] Didden R. (2007). “Effectieve behandeling van jeugdigen en volwassenen met een lichte verstandelijke beperking: een beschouwing [Effective treatment of adolescents and adults with a mild intellectual disability: a review],” in Met het oog op behandeling: effectieve behandeling van gedragsstoornissen van mensen met een licht verstandelijke beperking, eds R. Didden and X. Moonen (Utrecht: Landelijk Kenniscentrum LVG), 129–135.

[B51] Dunphy K. Baker F. A. Dumaresq E. Carroll-Haskins K. Eickholt J. Ercole M.. (2019). Creative arts interventions to address depression in older adults: a systematic review of outcomes, processes, and mechanisms. Front. Psychol. 9:2655. 10.3389/fpsyg.2018.02655 30671000 PMC6331422

[B52] Elliott R. (2010). Psychotherapy change process research: realizing the promise. Psychother. Res. 20, 123–135. 10.1080/10503300903470743 20099202

[B53] Elliott R. Greenberg L. S. Watson J. C. Timulak L. Freire E. (2013). “Research on humanistic-experiential psychotherapies,” in Bergin and Garfield's Handbook of Psychotherapy and Behavior Change, 6th Edn., ed M. J. Lambert (New York, NY: Wiley), 495–538.

[B54] Elliott R. (2012). “Qualitative methods for studying psychotherapy change processes,” in Qualitative Research Methods in Mental Health and Psychotherapy: A Guide for Students and Practitioners, eds A. Thompson and D. Harper (New York, NY: John Wiley and Sons), 69–81.

[B55] Ellis R. (2001). Movement metaphor as mediator: a model for the dance/movement therapy process. Arts Psychother. 28, 181–190. 10.1016/S0197-4556(01)00098-3

[B56] Fancourt D. Finn S. (2019). What Is the Evidence on the Role of the Arts in Improving Health and Well-Being? A Scoping Review. World Health Organization. Available online at: https://apps.who.int/iris/bitstream/handle/10665/329834/9789289054553-eng.pdf 32091683

[B57] Feniger-Schaal R. Hart Y. Lotan N. Koren-Karie N. Noy L. (2018). The body speaks: using the mirror game to link attachment and non-verbal behavior. Front. Psychol. 9:1560. 10.3389/fpsyg.2018.01560 30190699 PMC6115809

[B58] Feniger-Schaal R. Orkibi H. (2020). Integrative systematic review of drama therapy intervention research. Psychol. Aesthetics Creativity Arts 14, 68–80. 10.1037/aca0000257

[B59] Fish B. J. (2008). Formative evaluation of art-based supervision in art therapy training. Art Ther. 25, 70–77. 10.1080/07421656.2008.10129410

[B60] Flanagan C. S. (2004). Creative arts therapy in the rehabilitation of chronic pain; movement and metaphor–reflections by clients and therapist. Nordisk Fysioterapi 8, 120–131.

[B61] Frazier P. A. Tix A. P. Barron K. E. (2004). Testing moderator and mediator effects in counseling psychology research. J. Counsel. Psychol. 51, 115–134. 10.1037/0022-0167.51.1.115

[B62] Fuchs T. Koch S. C. (2014). Embodied affectivity: on moving and being moved. Front. Psychol. 5:508. 10.3389/fpsyg.2014.00508 24936191 PMC4047516

[B63] Gabel A. Robb M. (2017). (Re) considering psychological constructs: a thematic synthesis defining five therapeutic factors in group art therapy. Arts Psychother. 55, 126–135. 10.1016/j.aip.2017.05.005

[B64] Gaines A. M. Butler J. D. Holmwood C. (2015). Between drama education and drama therapy: international approaches to successful navigation. Performance 2.

[B65] Gardstrom S. C. Klemm A. Murphy K. M. (2017). Women's perceptions of the usefulness of group music therapy in addictions recovery. J. Music Ther. 26, 338–358. 10.1080/08098131.2016.1239649

[B66] Gauthier G. Guérin A. Zhdanava M. Jacobnson W. Nomikos Merikle E.. (2017). Treatment patterns, healthcare resource utilization, and costs following first-line antidepressant treatment in major depressive disorder: a retrospective US claims database analysis. BMC Psychiatry 17:222. 10.1186/s12888-017-1385-0 28629442 PMC5477263

[B67] Gelo O. C. G. Pritz A. Rieken B. (2015). Psychotherapy Research: Foundations, Process, and Outcome. Vienna: Springer.

[B68] Gerber N. (2006). “The therapist artist: individual and collective worldview,” in Essays on Identity by Art Therapists: Personal and Professional Perspectives, ed M. Junge (Springfield, IL: Charles C. Thomas), 85–94

[B69] Goldberg F. S. McNeil D. E. Binder R. (1988). Therapeutic factors in two forms of inpatient group psychotherapy: music therapy and verbal therapy. Group 12, 145–156. 10.1007/BF01456564

[B70] Goldstein J. A. (1971). Investigation of doubling as a technique for involving severely withdrawn patients in group psychotherapy. J. Consul. Clin. Psychol. 37, 155–162. 10.1037/h0020199 5565619

[B71] Grawe K. (1997). Research-informed psychotherapy. Psychother. Res. 7, 1–19. 10.1080/10503309712331331843

[B72] Grocke D. E. (2019). Guided Imagery and Music: The Bonny Method and Beyond, 2nd Ed. New Braunfels: Barcelona Publishers.

[B73] Haeyen S. Van Hooren S. Hutschemaekers G. (2015). Perceived effects of art therapy in the treatment of personality disorders, cluster B/C: a qualitative study. Arts Psychother. 45, 1–10. 10.1016/j.aip.2015.04.005

[B74] Hahna N. (2013). Towards an emancipatory practice: incorporating feminist pedagogy in the creative arts therapies. Arts Psychother. 40, 436–440. 10.1016/j.aip.2013.05.002

[B75] Hannigan S. Grima-Farrell C. Wardman N. (2019). Drawing on creative arts therapy approaches to enhance inclusive school cultures and student wellbeing. Issues Educ. Res. 29, 756–773.

[B76] Hardy G. E. Llewelyn S. (2015). “Introduction to psychotherapy process research,” in Psychotherapy Research: Foundations, Process, and Outcome, eds O. C. G. Gelo, A. Pritz, and B. Rieken (Vienna: Springer), 183–194.

[B77] Harvey S. Wang S. Kelly E. C. Wittig J. Li X. Peng X.. (2020). Creative dialogues across countries and culture during COVID-19. Creative Arts in Educ. Ther. 6, 72–84. 10.15212/CAET/2020/6/13

[B78] Hayes A. F. (2013). Introduction to Mediation, Moderation, and Conditional Process Analysis: A Regression-Based Approach. New York, NY: Guilford Press.

[B79] Heynen E. Roest J. Willemars G. van Hooren S. (2017). Therapeutic alliance is a factor of change in arts therapies and psychomotor therapy with adults who have mental health problems. Arts Psychother. 55, 111–115. 10.1016/j.aip.2017.05.006

[B80] Hilbuch A. Snir S. Regev D. Orkibi H. (2016). The role of art materials in the transferential relationship: art psychotherapists' perspective. Arts Psychother. 49, 19–26. 10.1016/j.aip.2016.05.011

[B81] Ho R. T. H. Fong T. C. T. Yip P. S. F. (2018). Perceived stress moderates the effects of a randomized trial of dance movement therapy on diurnal cortisol slopes in breast cancer patients. Psychoneuroendocrinology 87, 119–126. 10.1016/j.psyneuen.2017.10.012 29059542

[B82] Horvath A. O. Greenberg L. S. (1989). Development and validation of the working alliance inventory. J. Counsel. Psychol. 36, 223–233. 10.1037/0022-0167.36.2.22329733745

[B83] Imus S. D. (2021). “Creating breeds creating,” in Dance and Creativity Within Dance Movement Therapy: International Perspectives, eds H. Wengrower and S. Chaiklin (New York, NY: Routledge), 124–140.

[B84] Johnson D. R. (1998). On the therapeutic action of the creative arts therapies: the psychodynamic model. Arts Psychother. 25, 85–99. 10.1016/S0197-4556(97)00099-3

[B85] Jones P. (2007). Drama as Therapy: Theory, Practice, And Research, 2nd Edn. New York, NY: Routledge.

[B86] Jones P. (2021). Arts Therapies: A Revolution in Healthcare, 2nd Edn. New York, NY: Routledge.

[B87] Julliard K. Gujral J. K. Hamil S. Oswald E. Smyk A. Testa N. (2000). Art-based evaluation in research education. Art Ther. 17, 118–124. 10.1080/07421656.2000.10129513

[B88] Kaimal G. (2019). Adaptive Response Theory (ART): a clinical research framework for art therapy. Art Ther. 36, 215–219. 10.1080/07421656.2019.1667670

[B89] Kaimal G. Metzl E. Millrod E. (2017). Facilitative leadership: a framework for the creative arts therapies. Art Ther. 34, 146–151. 10.1080/07421656.2017.1343072

[B90] Kalaf L. Plante P. (2019). The lived experience of young syrian refugees with an expressive arts workshop about resilience. Can. Art Ther. Assoc. J. 32, 18–30. 10.1080/08322473.2019.1600895

[B91] Karkou V. Aithal S. Zubala A. Meekums B. (2019). Effectiveness of dance movement therapy in the treatment of adults with depression: a systematic review with meta-analyses. Front. Psychol. 10:936. 10.3389/fpsyg.2019.00936 31130889 PMC6509172

[B92] Karkou V. Sanderson P. (eds.). (2006). Arts Therapies: A Research-Based Map of the Field. Edinburgh: Elsevier.

[B93] Kazdin A. E. (2007). Mediators and mechanisms of change in psychotherapy research. Annu. Rev. Clin. Psychol. 3, 1–27. 10.1146/annurev.clinpsy.3.022806.091432 17716046

[B94] Kazdin A. E. (2009). Understanding how and why psychotherapy leads to change. Psychother. Res. 19, 418–428. 10.1080/10503300802448899 19034715

[B95] Kedem-Tahar E. Kellermann P. F. (1996). Psychodrama and drama therapy: a comparison. Arts Psychother. 23, 27–36. 10.1016/0197-4556(95)00059-3

[B96] Keidar L. Snir S. Regev D. Orkibi H. Adoni-Kroyanker M. (2020). Relationship between the therapist-client bond and outcomes of art therapy in the Israeli school system. Art Ther. 1–8. 10.1080/07421656.2020.1827651

[B97] Kellermann P. F. (1985). Participants' perception of therapeutic factors in psychodrama. J. Group Psychother. Psychodrama Sociometry 38, 123–132.

[B98] Kellermann P. F. (1987). Psychodrama participants' perception of therapeutic factors. Small Group Res. 18, 408–419. 10.1177/104649648701800307

[B99] Kellet S. Hall J. Compton Dickinson S. (2019). Group cognitive analytic music therapy: a quasi-experimental feasibility study conducted in a high secure hospital. Nordic J. Music Ther. 28, 224–255. 10.1080/08098131.2018.1529697

[B100] Kestenberg Amighi J. Loman S. Sossin K. M. (2018). The Meaning of Movement: Embodied Developmental, Clinical, and Cultural Perspectives of the Kestenberg Movement Profile. New York, NY: Routledge.

[B101] Kim K. W. (2003). The effects of being the protagonist in psychodrama. *J*. Group Psychother. Psychodrama Sociometry 55, 115–127. 10.3200/JGPP.55.4.115-127

[B102] Knight A. Matney B. (2014). Percussion pedagogy: a survey of music therapy faculty. Music Ther. Perspect. 32, 109–115. 10.1093/mtp/miu010

[B103] Koch S. C. (2014). Rhythm is it: effects of dynamic body feedback on affect and attitudes. Front. Psychol. 5:537. 10.3389/fpsyg.2014.00537 24959153 PMC4051267

[B104] Koch S. C. (2017). Arts and health: active factors in arts therapies and a theory framework of embodied aesthetics. Arts Psychother. 54, 85–91. 10.1016/j.aip.2017.02.002

[B105] Koch S. C. Fischman D. (2011). Embodied enactive dance movement therapy. Am. J. Dance Ther. 33, 57–72. 10.1007/s10465-011-9108-4

[B106] Koch S. C. Fuchs T. (2011). Embodied arts therapies. Arts Psychother. 38, 276–280. 10.1016/j.aip.2011.08.007

[B107] Koch S. C. Mergheim K. Raeke J. Machado C. B. Riegner E. Nolden J.. (2016). The embodied self in Parkinson's disease: feasibility of a single tango intervention for assessing changes in psychological health outcomes and aesthetic experience. Front. Neurosci. 10:287. 10.3389/fnins.2016.00287 27458332 PMC4935674

[B108] Koch S. C. Morlinghaus K. Fuchs T. (2007). The joy dance: specific effects of a single dance intervention on psychiatric patients with depression. Arts Psycho Ther. 34, 340–349. 10.1016/j.aip.2007.07.001

[B109] Koch S. C. Riege R. F. F. Tisborn K. Biondo J. Martin L. Beelmann A. (2019). Effects of dance movement therapy and dance on health-related psychological outcomes. A meta-analysis update. Front. Psychol. 10:1806. 10.3389/fpsyg.2019.01806 31481910 PMC6710484

[B110] Koelsch S. (2015). Music-evoked emotions: principles, brain correlates, and implications for therapy. Ann. N. Y. Acad. Sci. 1337, 193–201. 10.1111/nyas.12684 25773635

[B111] Kushnir A. Orkibi H. (2021). Concretization as a mechanism of change in psychodrama: procedures and benefits. Front. Psychol. 12:633069. 10.3389/fpsyg.2021.633069 33708162 PMC7940662

[B112] LaGasse A. B. Hickle T. (2015). Perception of community and learning in a distance and resident graduate course. Music Ther. Perspect. 35, 79–87. 10.1093/mtp/miv027

[B113] Lambert M. J. (2013). “The efficacy and effectiveness of psychotherapy,” in Bergin and Garfield's Handbook of Psychotherapy and Behavior Change, 6th Edn. (Hoboken, NJ: John Wiley), 169–218.

[B114] Landis-Shack N. Heinz A. J. Bonn-Miller M. O. (2017). Music therapy for postraumatic stress in adults: a theoretical review. Psychomusicology 27, 334–342. 10.1037/pmu0000192 29290641 PMC5744879

[B115] Landy R. (1997). Drama therapy and distancing: reflections on theory and clinical application. Arts Psychother. 23, 367–373. 10.1016/S0197-4556(96)00052-4

[B116] Landy R. J. Hodermarska M. Mowers D. Perrin D. (2012). Performance as art-based research in drama therapy supervision. J. Appl. Arts Health 3, 49–58. 10.1386/jaah.3.1.49_1

[B117] Landy R. J. McLellan L. McMullian S. (2005). The education of the drama therapist: in search of a guide. Arts Psychother. 32, 275–292. 10.1016/j.aip.2005.02.005

[B118] Lange G. Leonhart R. Gruber H. Koch S. C. (2018). The effect of active creation on psychological health: a feasibility study on (therapeutic) mechanisms. Behav. Sci. 8:25. 10.3390/bs8020025 29439541 PMC5836008

[B119] Lauffenberger S. K. (2020). ‘Something more:' the unique features of dance movement therapy/psychotherapy. Am. J. Dance Ther. 42, 16–32. 10.1007/s10465-020-09321-y

[B120] Lee C. (2000). A method of analyzing improvisations in music therapy. J. Music Ther. 37, 147–167. 10.1093/jmt/37.2.147 10979757

[B121] Lusebrink V. B. Mārtinsone K. Dzilna-Šilova I. (2013). The expressive therapies continuum (ETC): interdisciplinary bases of the ETC. Int. J. Art Ther. 18, 75–85. 10.1080/17454832.2012.713370

[B122] MacDonald R. Kreutz G. Mitchell L. (eds.). (2013). Music, Health, and Wellbeing. Oxford: Oxford University Press.

[B123] Mannheim E. G. Helmes A. Weis J. (2013). Dance/movement therapy in oncological rehabilitation. Forsch Komplementmed 20, 33–41. 10.1159/000346617 23727761

[B124] McCaw D. (ed.). (2012). The Laban Sourcebook. London: Routledge.

[B125] McDermott O. Crellin N. Ridder H. M. Orrell M. (2013). Music therapy in dementia: a narrative synthesis systematic review. Int. J. Geriatric Psychiatry 28, 781–794. 10.1002/gps.3895 23080214

[B126] McLeod B. D. Weisz J. R. (2005). The therapy process observational coding system-alliance scale: measure characteristics and prediction of outcome in usual clinical practice. J. Consul. Clin. Psychol. 73, 323–333. 10.1037/0022-006X.73.2.323 15796640

[B127] Meekums B Karkou V Nelson A. (2015). Dance movement therapy for depression. Cochrane Database Syst. Rev. CD009895. 10.1002/14651858.CD009895.pub2 PMC8928931 25695871

[B128] Meekums B. (2002). Dance Movement Therapy: A Creative Psychotherapeutic Approach. Thousand Oaks, CA: Sage Publications.

[B129] Millet C. R. Gooding L. F. (2017). Comparing active and passive distraction-based music therapy interventions on preoperative anxiety in pediatric patients and their caregivers, J. Music Ther. 54, 460–478. 10.1093/jmt/thx014 29253180

[B130] Moon B. (2003). Essentials of Art Therapy Education and Practice. Springfield, IL: Charles C Thomas.

[B131] Moreno J. L. (1965). Therapeutic vehicles and the concept of surplus reality. Group Psychother. 18, 211–216.

[B132] Moreno J. L. (1972/1994). Psychodrama - First Volume: Psychodrama and Group Psychotherapy, 4th Edn. Mclean, VA: American Society of Group Psychotherapy and Psychodrama.

[B133] Morgan-Lopez A. A. MacKinnon D. P. (2006). Demonstration and evaluation of a method for assessing mediated moderation. Behav. Res. Methods 38, 77–87. 10.3758/BF03192752 16817516

[B134] Munn Z. Peters M. Stern C. Tufanaru C. McArthur A. Aromataris E. (2018). Systematic review or scoping review? Guidance for authors when choosing between a systematic or scoping review approach. BMC Med. Rese. Methodol. 18:143. 10.1186/s12874-018-0611-x 30453902 PMC6245623

[B135] Nitzan A. Orkibi H. (2020). Stigma correlates in individuals with mental health conditions versus community members enrolled in a nationwide integrated arts-based community rehabilitation program in Israel. Health Social Care Commun. 28, 1230–1240. 10.1111/hsc.12956 32052530

[B136] Nock M. K. (2007). Conceptual and design essentials for evaluating mechanisms of change. Alcoholism 31, 4s–12s. 10.1111/j.1530-0277.2007.00488.x 17880341

[B137] Nolan E. (2019). Opening art therapy thresholds: mechanisms that influence change in the community art therapy studio. Art Ther. 36, 77–85. 10.1080/07421656.2019.1618177

[B138] Norcross J. C. (2011). Psychotherapy Relationships That Work: Evidence-Based Responsiveness, 2nd Edn. Oxford: Oxford University Press.

[B139] Nordoff P. Robbins C. (1966). The Three Bears. Pitsburgh, PA: Theodore Presser Company.

[B140] Oezbay H. Goeka E. Oeztuerk E. Guengoer S. Hincal G. (1993). Therapeutic factors in an adolescent psychodrama group. J. Group Psychother. Psychodrama Sociometry 46, 3–11.

[B141] Orkibi H. (2012). A field training model for creative arts therapists: report from a 3-year program evaluation. Art Ther. 29, 174–179. 10.1080/07421656.2012.730930

[B142] Orkibi H. (2019). Positive psychodrama: a framework for practice and research. Arts Psychother. 66:101603. 10.1016/j.aip.2019.101603

[B143] Orkibi H. (2020). Creative Arts Therapies. The Society for the Psychology of Aesthetics, Creativity, and the Arts - Division 10 of the American Psychological Association. Available online at: http://www.div10.org/creative-arts-therapies

[B144] Orkibi H. (2021). Creative adaptability: conceptual framework, measurement, and outcomes in times of crisis. Front. Psychol. 11:588172. 10.3389/fpsyg.2020.588172 33510671 PMC7835130

[B145] Orkibi H. Azoulay B. Regev D. Snir S. (2017a). Adolescents' dramatic engagement predicts their in-session productive behaviors: a psychodrama change process study. Arts Psychother. 55, 46–53. 10.1016/j.aip.2017.04.001

[B146] Orkibi H. Azoulay B. Snir S. Regev D. (2017b). In-session behaviours and adolescents' self-concept and loneliness: a psychodrama process–outcome study. Clin. Psychol. Psychother. 24, O1455–O1463. 10.1002/cpp.2103 28653318

[B147] Orkibi H. Bar N. Eliakim I. (2014). The effect of drama-based group therapy on aspects of mental illness stigma. Arts Psychother. 41, 458–466. 10.1016/j.aip.2014.08.006

[B148] Orkibi H. Biancalani G. Bucutã M. D. Sassu R. Wieser M. A. Franchini L.. (2021). Students' confidence and interests in palliative and bereavement care: a European study. Front. Psychol. 12:616526. 10.3389/fpsyg.2021.616526 33679532 PMC7930718

[B149] Orkibi H. Feniger-Schaal R. (2019). Integrative systematic review of psychodrama psychotherapy research: trends and methodological implications. PLoS ONE 14:e0212575. 10.1371/journal.pone.0212575 30779787 PMC6380607

[B150] Pallaro P. (ed.). (2007). Authentic Movement: Moving the Body, Moving the Self, Being Moved: A Collection of Essays-Volume Two. London: Jessica Kingsley Publishers.

[B151] Parsons A. Omylinska-Thurston J. Karkou V. Harlow J. Haslam S. Hobson J.. (2020). Arts for the blues – a new creative psychological therapy for depression. Br. J. Guidance Counsel. 48, 5–20. 10.1080/03069885.2019.1633459 30950682

[B152] Passiali V. (2012). Supporting parent-child interactions: music therapy as an intervention for promoting mutually responsive orientation. J. Music Ther. 49, 303–334. 10.1093/jmt/49.3.303 23259232

[B153] Payne H. Koch S. Tantia J. (eds.). (2019). The Routledge International Handbook of Embodied Perspectives in Psychotherapy: Approaches From Dance Movement and Body Psychotherapies. London: Routledge.

[B154] Pendzik S. (2006). On dramatic reality and its therapeutic function in drama therapy. Arts Psychother. 33, 271–280. 10.1016/j.aip.2006.03.001

[B155] Peters M. D. Godfrey C. M. Khalil H. McInerney P. Parker D. Soares C. B. (2015). Guidance for conducting systematic scoping reviews. Int. J. Evidence Based Healthc. 13, 141–146. 10.1097/XEB.0000000000000050 26134548

[B156] Pilgrim K. Ventura N. Bingen A. Faith E. Fort J. Reyes O.. (2020). From a distance: technology and the first low-residency drama therapy education program. Drama Ther. Rev. 6, 27–48. 10.1386/dtr_00014_1

[B157] Porter S. McConnell T. Clarke M. Kirkwood J. Hughes N. Graham-Wisner L.. (2017). A critical realist evaluation of a music therapy intervention in palliative care. BMC Palliative Care 16, 1–12. 10.1186/s12904-017-0253-5 29221475 PMC5723094

[B158] Potvin N. Bradt J. Ghetti C. (2018). A theoretical model of resource-oriented music therapy with informal hospice caregivers during pre-bereavement. J. Music Ther. 55, 27–61. 10.1093/jmt/thx019 29438566

[B159] Preuss A. Bolliger B. A. Schicho J. Hättenschwiler J. Seifritz E. Bruehl A. B.. (2020). SSRI treatment response prediction in depression based on brain activation by emotional stimuli. Front. Psychiatry 11:1071. 10.3389/fpsyt.2020.538393 33281635 PMC7691246

[B160] Pylvänäinen P. (2018). Dance Movement Therapy in the Treatment of Depression: Change in Body Image and Mood-a Clinical Practice-based Study. Jyväskylä Studies in Education, Psychology and Social Research.

[B161] Pylvänäinen P. M. Muotka J. S. Lappalainen R. (2015). A dance movement therapy group for depressed adult patients in a psychiatric outpatient clinic: effects of the treatment. Front. Psychol. 6:980. 10.3389/fpsyg.2015.00980 26217292 PMC4498018

[B162] Quinlan R. Schweitzer R. D. Khawaja N. Griffin J. (2016). Evaluation of a school-based creative arts therapy programme for adolescents from refugee backgrounds. Arts Psychother. 47, 72–78. 10.1016/j.aip.2015.09.006

[B163] Ramsayer F. Tschacher W. (2011). Nonverbal synchrony in psychotherapy: coordinated body movement reflects relationship quality and outcome. J. Consul. Clin. Psychol. 79, 284–295. 10.1037/a0023419 21639608

[B164] Ram-Vlasov N. Orkibi H. (2021). The kinetic family in action: an intermodal assessment model. Arts Psychother. 71:101750. 10.1016/j.aip.2020.101750

[B165] Robb S. L. (2000). The effect of therapeutic music interventions on the behavior of hospitalized children in isolation: developing a contextual support model of music therapy. J. Music Ther. 37, 118–146. 10.1093/jmt/37.2.118 10932125

[B166] Rolvsjord R. I. (2010). What clients do to make music therapy work: a qualitative multiple case study in adult mental health care. Nordic J. Music Ther. 24, 296–321. 10.1080/08098131.2014.964753

[B167] Rosenzweig S. (1936). Some implicit common factors in diverse methods of psychotherapy. Am. J. Orthopsychiatry 6, 412–415. 10.1111/j.1939-0025.1936.tb05248.x

[B168] Rush A. J. Fava M. Wisniewski S. R. Lavori P. W. Trivedi M. H. Sackeim H. A.. (2004). Sequenced treatment alternatives to relieve depression (STAR*D): Rationale and design. Control. Clin. Trials, 25, 119–142. 10.1016/s0197-2456(03)00112-0 15061154

[B169] Rylatt P. (2012). The benefits of creative therapy for people with dementia. Nursing Standard 26, 42–47. 10.7748/ns.26.33.42.s50 22616268

[B170] Sajnani N. Beardall N. Chapin Stephenson R. Estrella K. Zarate R. Socha B.. (2019). “Navigating the transition to online education in the arts therapies,” in Ecarte Conference Proceedings 2017, eds R. Houghham, S. Pitruzella, and S. Scoble (Plymouth, MA: University of Plymouth Press).

[B171] Sajnani N. Meyer C. Tillberg-Webb H. (2020). Aesthetic presence: the role of the arts in the education of creative arts therapies. Arts Psychother. 69, 1–9. 10.1016/j.aip.2020.101668 PMC7245298 32501320

[B172] Sandel S. L. Chaiklin S. Lohn A. (1993). Foundations of dance movement therapy: the life and work of Marian Chace. Marian Chace Memorial Fund Am. Dance Ther. Assoc.

[B173] Schiltz L. (2014). Multimodal arts psychotherapy with adolescents suffering from conduct disorders. Arts Psychother. 41, 187–192. 10.1016/j.aip.2014.02.005

[B174] Schmais C. (1985). Healing processes in group dance therapy. Am. J. Dance Ther. 8, 17–36. 10.1007/BF02251439

[B175] Shim M. (2015). Factors and mechanisms of dance/movement therapy for resilience-building in people living with chronic pain. J. Pain 16:S111. 10.1016/j.jpain.2015.01.462

[B176] Shim M. Goodill S. W. Bradt J. (2019). Mechanisms of dance/movement therapy for building resilience in people experiencing chronic pain. Am. J. Dance Ther. 41, 87–112. 10.1007/s10465-019-09294-7

[B177] Shim M. Johnson R. B. Gasson S. Goodill S. Jermyn R. Bradt J. (2017). A model of dance/movement therapy for resilience-building in people living with chronic pain. Euro. J. Integr. Med. 9, 27–40. 10.1016/j.eujim.2017.01.011

[B178] Short A. Gibb H. Holmes C. (2009). Integrating words, images, and text in BMGIM: finding connections through semiotic intertextuality. Nordic J. Music Ther. 20, 3–21. 10.1080/08098131003764031

[B179] Shuper-Engelhard E. Shacham M. Vulcan M. (2019). Clinical intervention using dance-movement psychotherapy for couples: qualitative research and clinical implications. Body Movement Dance Psychother. 14, 128–142. 10.1080/17432979.2019.1618395

[B180] Smith D. Wright C. J. Lakhani A. Zeeman H. (2017). Art processes: a research tool for acquired brain injury and residential design. Arts Health 9, 251–268. 10.1080/17533015.2017.1354899

[B181] Sonke J. Sams K. Morgan-Daniel J. Schaefer N. Pesata V. Golden T.. (2021). Health communication and the arts in the united states: a scoping review. Am. J. Health Promotion 35, 106–115. 10.1177/0890117120931710 32551833

[B182] Sucharew H. Macaluso M. (2019). Methods for research evidence synthesis: the scoping review approach. J. Hospital Med. 14, 416–418. 10.12788/jhm.3248 31251164

[B183] Testoni I. Cecchini C. Zulian M. Guglielmin M. S. Ronconi L. Kirk K.. (2018). Psychodrama in therapeutic communities for drug addiction: a study of four cases investigated using idiographic change process analysis. Arts Psychother. 61, 10–20. 10.1016/j.aip.2017.12.007

[B184] Thaut M. H. Hoemberg V. (2014). Handbook of Neurologic Music Therapy. New York, NY: Oxford University Press.

[B185] Thaut M. H. Kenyon G. P. Schauer M. L. McIntosh G. C. (1999). The connection between rhythmicity and brain function. IEEE Eng. Med. Biol. Mag. 18, 101–108. 10.1109/51.752991 10101675

[B186] Thaut M. H. McIntosh G. C. Hoemberg V. (2015). Neurobiological foundations of neurologic music therapy: rhythmic entrainment and the motor system. Front. Psychol. 5:1185. 10.3389/fpsyg.2014.01185 25774137 PMC4344110

[B187] Thomas D. R. (2003). A General Inductive Approach for Qualitative Data Analysis. Auckland: School of Population Health, University of Auckland.

[B188] Thomas D. R. (2006). A general inductive approach for analyzing qualitative evaluation data. Am. J. Evaluat. 27, 237–246. 10.1177/1098214005283748

[B189] Timulak L. (2008). Research in Psychotherapy and Counselling. London: SAGE.

[B190] Tricco A. C. Lillie E. Zarin W. O'Brien K. Colquhoun H. Kastner M.. (2016). A scoping review on the conduct and reporting of scoping reviews. BMC Med. Res. Methodol. 16:15. 10.1186/s12874-016-0116-4 26857112 PMC4746911

[B191] van der Merwe J. (2020). Change Process Research and the Common Factors Approach in Conceptualising Psychotherapeutic Change: A Systematic Review. Johannesburg: North-West University.

[B192] Van Lith T. (2015). Art making as a mental health recovery tool for change and coping. Art Ther. 32, 5–12. 10.1080/07421656.2015.992826

[B193] Van Lith T. (2016). Art therapy in mental health: a systematic review of approaches and practices. Arts Psychother. 47, 9–22. 10.1016/j.aip.2015.09.003

[B194] Vega V. P. Keith D. (2012). A survey of online courses in music therapy. Music Ther. Perspect. 30, 176–182. 10.1093/mtp/30.2.176

[B195] Ventouras E. C. Margariti A. Chondraki P. Kalatzis I. Economou N. T. Tsekou H.. (2015). EEG-based investigation of brain connectivity changes in psychotic patients undergoing the primitive expression form of dance therapy: a methodological pilot study. Cogn. Neurodyn. 9, 231–248. 10.1007/s11571-014-9319-8 25852781 PMC4378579

[B196] Von Elm E. Schreiber G. Haupt C. (2019). Methodische Anleitung für Scoping Reviews [Methodological Manual for Scoping Reviews]. Zeitschrift für Evidenz, Fortbildung und Qualität im Gesundheitswesen 143, 1–7. 10.1016/j.zefq.2019.05.004 31296451

[B197] Waldon E. G. (2001). The effects of group music therapy on mood states and cohesiveness in adult oncology patients. J. Music Ther. 38, 212–238. 10.1093/jmt/38.3.212 11570933

[B198] Wampold B. E. (2001). The Great Psychotherapy Debate: Models, Methods, and Findings. Mahwah, NJ: Erlbaum.

[B199] Wampold B. E. (2015). How important are the common factors in psychotherapy? An update. World Psychiatry 14, 270–277. 10.1002/wps.20238 26407772 PMC4592639

[B200] Wampold B. E. Irmel Z. E. (2015). The Great Psychotherapy Debate: The Evidence for What Makes Psychotherapy Work, 2nd Edn. New York, NY: Routledge.

[B201] Warth M. Kessler J. Hillecke T. Bardenheuer H. J. (2016). Trajectories of terminally ill patients' cardiovascular response to receptive music therapy in palliative car. J. Pain Symptom Manage. 52, 196–204. 10.1016/j.jpainsymman.2016.01.008 27090850

[B202] Wiedenhofer S. Koch S. C. (2017). Active factors in dance/movement therapy: specifying health effects of non-goal-orientation in movement. Arts Psychother. 52, 10–23. 10.1016/j.aip.2016.09.004

[B203] Winnicott D. W. (1980). Playing and Reality. London: Tavistock Publications.

[B204] Winther H. Stelter R. (2008). Gypsy or hedgehog? Movement, energy and change in dance therapeutic processes in Dansergia. Body Movement Dance Psychother. 3, 45–56, 10.1080/17432970701664506

[B205] Wosch T. Wigram T. (2007). “Microanalysis in music therapy: introduction and theoretical basis,” in Microanalysis: Methods, Techniques and Applications for Clinicians, Researchers, Educators and Students (London: Jessica Kingsley), 13–28.

[B206] Yalom I. D. (1983). Inpatient Group Psychotherapy. New York, NY: Basic Books.

[B207] Yalom I. D. (1995). The Theory And practice of Group Psychotherapy, 4th Edn. Washington, DC: American Psychological Association.

[B208] Young J. L. (2012). Bringing my body into my body of knowledge as a dance/movement therapy educator. Am. J. Dance Ther. 34, 141–158. 10.1007/s10465-012-9137-7

