# Supplementary material for: From Therapeutic Factors to Mechanisms of Change in the Creative Arts Therapies: A Scoping Review
Source: Front Psychol. 2021 Jul 15;12:678397. doi: 10.3389/fpsyg.2021.678397 (PMC8336579; doi:10.3389/fpsyg.2021.678397)
Supplement: Supplementary file 1 [file Data_Sheet_1.PDF]

From Therapeutic Factors to Mechanisms of Change in the Creative Arts Therapies: A Scoping Review

Martina de Witte, Hod Orkibi, Rebecca Zarate, Vicky Karkou, Nisha Sajnani,

Bani Malhotra, Rainbow Tin Hung Ho, Girija Kaimal, Felicity A. Baker, & Sabine C. Koch

Online Supplementary Materials File

Page	Content
2	Exemplary of Search String for the PsycINFO Database
3	Table S1 - *Therapeutic Factors of Art Therapy*
7	Table S2 - *Therapeutic Factors of Dance Movement Therapy*
14	Table S3 - *Therapeutic Factors of Drama Therapy and Psychodrama*
17	Table S4 - *Therapeutic Factors of Music Therapy*
22	Table S5 - *Therapeutic Factors in Studies with More than One CAT Modality*

Exemplary Search String for the PsycINFO Database

S1DE "Creative Arts Therapy" OR TI ((Expressive OR arts OR artistic) W0 (therap* OR psychotherap* OR treatment* OR intervention* OR program*)) OR AB ((Expressive OR arts OR artistic) W0 (therap* OR psychotherap* OR treatment* OR intervention* OR program*)) OR SU ((Expressive OR arts OR artistic) W0 (therap* OR psychotherap* OR treatment* OR intervention* OR program*))

S2DE "Art Therapy" OR TI (art N1 (therap* OR psychotherap* OR treatment* OR intervention* OR program*)) OR AB (art N1 (therap* OR psychotherap* OR treatment* OR intervention* OR program*)) OR SU (art N1 (therap* OR psychotherap* OR treatment* OR intervention* OR program*))

S3TI ((dance OR dancing) N3 (therap* OR psychotherap* OR treatment* OR intervention* OR program*)) OR AB ((dance OR dancing) N3 (therap* OR psychotherap* OR treatment* OR intervention* OR program*)) OR SU ((dance OR dancing) N3 (therap* OR psychotherap* OR treatment* OR intervention* OR program*))

S4DE "Psychodrama" OR TI (psychodrama OR (psycho N1 drama*) OR (drama N1 therap*) OR dramatherap*) OR AB (psychodrama OR (psycho N1 drama*) OR (drama N1 therap*) OR dramatherap*)) OR SU (psychodrama OR (psycho N1 drama*) OR (drama N1 therap*) OR dramatherap*))

S5DE "Music Therapy" OR TI (Music* N3 (therap* OR psychotherap* OR treatment* OR intervention* OR program*)) OR AB (Music* N3 (therap* OR psychotherap* OR treatment* OR intervention* OR program*)) OR SU (Music* N3 (therap* OR psychotherap* OR treatment* OR intervention* OR program*))

S6TI (mechanism* OR ((Therapeutic OR Healing OR active OR common OR change OR helpful) factor* or specific) N1 factor*) OR (Change N1 process*) OR (key N1 ingredients))

Table S1

Therapeutic Factors of Art Therapy (AT)

**Author(s)**	**Year**	**Study purpose**	***N***	**Study design**	**Participants / setting**	**Intervention characteristics**	**Therapeutic factors**	**Outcome(s)**
Abbing et al.	2018	Identify mechanisms to assess effectiveness of AT for anxiety in adults	3	Quan Systematic Review<br><br>(3 publications included)	Individuals with anxiety in outpatient settings (Universities and Prison)	AT (individual or group)	Nonverbal (JF)<br>Tactile quality (SF)<br>Flow state (JF)<br>Relaxation (JF)<br>Emotion Regulation (CF)<br>Safe environment (CF):<br>Cognitive Regulation (CF)<br>Reflection upon art (SF)<br>Insights in emotions (CF):<br>Unconscious self-expression (JF)	Anxiety
Bosgraaf et al.	2020	Identify art therapeutic components that contribute to the reduction of psychosocial problems in children and adolescents	37	Quan and Qual Systematic Review<br><br>(37 publications included)	Children and adolescents with psychosocial problems	AT (Individual or group)	Form of visual self-expression (SF)<br>Self-awareness through the artwork (SF)<br>Visual narrative of life (SF)<br>Artmaking in the session as a form of exploration and/or reflection (SF)<br>Use of specific art materials/techniques (SF)<br>To learn or practice artistic skills (JF)<br>Positive and safe intervention for developmental stage for children and adolescents (CF)<br>Group process (CF)<br>Therapeutic alliance (CF)	Self-concept;<br>Self-esteem;<br>Emotion regulation

Author(s)	Year	Study purpose	*N*	Study design	Participants / setting	Intervention characteristics	Therapeutic factors	Outcome(s)
							Art therapy as integrative activation of the brain*	
Czamanski-Cohen et al.	2019	Assess the role of emotional processing in art therapy (REPAT) for breast cancer patients	20	Quan<br>RCT	Outpatient Clinic-Breast Cancer patients in survivorship	AT: based on body-mind work (AT group- one and half hour; control group-one hour)	Emotion processing (CF)	Depression;<br>Somatic symptoms
Deboys et al.	2017	Identify/develop a model for processes of change in school-based art therapy	40	Qual<br>Grounded theory	Educational setting- 2 Primary Schools	AT individual	Expression (CF);<br>Expressing the non-verbal (JF)	Mood;<br>Confidence;<br>Communication;<br>Understanding
Gabel & Robb	2017	To develop definitions of group art therapeutic factors/ mechanisms of change	119	Qual<br>Thematic Meta-synthesis	Art therapy in group settings	AT group	Symbolic expression (JF);<br>Relational aesthetics (JF);<br>Embodiment (JF);<br>Pleasure/play (JF);<br>Rituals (JF)	-
Haeyen et al.	2015	Identify/develop model on effects of art therapy in the treatment of personality disorders	29	Qual<br>Grounded Theory	Adults with personality disorders	AT	Discovering materials and possibilities (SF);<br>Emotional reaction to materials (SF);<br>Portraying self-image (SF);<br>Portraying feelings of past/future (SF)<br>Seeing own emotions through visual art (SF);<br>Transcending thinking on product/process (JF);<br>Body awareness (JF);<br>Reflection on own patterns (CF)<br>Acting out (CF)	Change/coping from personality disorders, cluster B/C

Author(s)	Year	Study purpose	*N*	Study design	Participants / setting	Intervention characteristics	Therapeutic factors	Outcome(s)
							"Living through" emotions and directing this process (CF)<br>Awareness/exploring own feelings (CF);<br>Differentiating/clarifying feelings/thoughts (CF)<br>Experiencing the present moment (CF);<br>Improving feedback skills (CF)	
Hillbuch et al.	2016	Examine the role of art materials, art making, and the resulting artistic product in the transferential relationship	10	Qual<br>Grounded Theory	Art psycho-therapist	AT	Transference during artmaking (SF);<br>Transference to the artistic product (SF)	-
Keidar et al.	2020	Examine the associations between the therapist-client bond and outcomes in individual and group art therapy in the school system	127	Quan	Israeli education system	AT (individual or group)	Therapist-client bond (CF)	Self-perception internalized behavior problems, loneliness
Nolan	2019	Identify mechanisms that influence change in the community art therapy studio	3	Qual<br>Art-based phenomenological reduction	Art therapists in community settings	Community Group AT	Safety (CF);<br>Structure (JF);<br>Acceptance of self / others (CF);<br>Acceptance of art work (JF);<br>Opportunity to explore (JF)	Positive progression / development / growth in the community
Van Lith	2015	To provide evidence of how art making may help clients as a tool for change and coping in their recovery from mental illness.	12	Qual<br>Longitudinal, multiple case study	Adults with mental illness in recovery (2 Psychosocial rehabilitation organizations)	AT	Connection to inner self (CF);<br>Developing sense of achievement (CF);<br>Motivational force when unwell (CF);	Change / coping in recovery from mental illness

Author(s)	Year	Study purpose	*N*	Study design	Participants / setting	Intervention characteristics	Therapeutic factors	Outcome(s)
							Psychological safe space (CF)	

Note. CF = common factor of psychotherapies, JF = joint factor across CATs disciplines, SF = specific factor in a specific CATs discipline, Qual = qualitative study, Quan = quantitative study, RCT = randomized controlled trial.

Table S2

Therapeutic Factors of Dance Movement Therapy (DMT)

Author(s)	Year	Study purpose	*N*	Study design	Participants / setting	Intervention characteristics	Therapeutic factors	Outcome(s)
Chyle et al.	2020	Identifying therapeutic factors of body-movement interventions	23	Narrative review<br><br>(23 publications included)	Rehabilitation of male offenders	Body- and movement-based interventions (DMT and others)	1. Interacting with one another in movement (SF)<br>-Moving in synchronicity (SF)<br>-Encountering one another (SF)<br>-Establishing cooperation and (group) cohesion (CF)<br>-Moving with the therapist (SF)<br>2. Performative and creative factors (JF)<br>-Moving spontaneously (SF)<br>-Re-enactment of nonverbal behavioural patterns (JF)<br>-Self-display (JF)<br>-Changing and embodying of roles (SF)<br>-Designing of model situations and testing alternative behaviour (JF)<br>-Designing of group dynamic themes (JF)<br>-Actualising and performing biographic	9 bodily orientations:<br>1. interpersonal skills;<br>2. Emotional skills;<br>3. Physical skills;<br>4. Perceptual skills;<br>5. Dealing with life story;<br>6. Action skills;<br>7. Relating to self;<br>8. Skills in experience;<br>9. Cognitive skills

Author(s)	Year	Study purpose	*N*	Study design	Participants / setting	Intervention characteristics	Therapeutic factors	Outcome(s)
							and crime-specific themes (JF)<br>3. Physical factors (SF)<br>-Experiencing the body (SF)<br>-Mobilisation and vitalisation (JF)<br>-Focusing oneself (JF)<br>-Losing control (JF)<br>-Using bodily sensation as a source of information (SF)<br>4. Emotional factors (CF)<br>-Actualising emotions (CF)<br>-Expressing emotions (CF)<br>-Regulating emotions (CF)<br>-Transforming emotions (CF)<br>-Structuring emotional outlets (JF)<br>5. Confrontation as therapeutic factors (CF)<br>-Mastery of dynamic challenges (CF)<br>-Confronting oneself with emotions (JF)<br>-Confronting oneself with own actions (JF)<br>6. Reciprocal factors (CF)<br>-Remembering (CF)	

Author(s)	Year	Study purpose	*N*	Study design	Participants / setting	Intervention characteristics	Therapeutic factors	Outcome(s)
							-Establishing inner connections (CF)<br>-Connecting movement and language with one another (SF)<br>-Disclosing life issues and offences in movement (SF)<br>7. Physical factors (SF)<br>-Learning and practicing of motion sequences (SF)<br>-Skills training (JF)<br>-Participation in ritualised processes (JF)<br>8. Perceptual factors (CF)<br>-Self-exploration (CF)<br>-Perceiving emotions (CF)<br>-Perceiving own blockages (CF)	
Ellis	2001	Understanding how the movement metaphor can act as a mediator in the therapeutic process	4	Qual	Patients with schizophrenia/ in-patient rehabilitation unit	DMT	Movement Metaphor (SF):<br>-Shifting between the symbolic and the knowing realms (JF);<br>-Shifting between movement and meaning (SF).	N/A; focus on process
Ho et al.	2018	Investigation of effects of DMT on breast cancer, taking into	121	Quan moderated mediation analysis on the basis of RCT trial (Ho, 2016)	Acute oncology treatment (patients undergoing	DMT: 6 group sessions (2 per week),	Perceived stress (at baseline) (CF)	Distress (cortisol levels); Perceived stress (at posttest)

Author(s)	Year	Study purpose	*N*	Study design	Participants / setting	Intervention characteristics	Therapeutic factors	Outcome(s)
		account the stress level at baseline as a mediator			radiation therapy)	accompanying radiation therapy		
Koch et al.	2007	Assessing effects of bouncing (up and down) movement on well-being in depressed patients	31	Quan<br>RCT with 3 arms: circle dance intervention vs. listening to just music vs. riding ergo- bike	Inpatient psychiatry, depressed adult patients	DMT group session: one-time-intervention (1h) using jumping rhythm	Jumping rhythm (Bouncing) (SF);<br>-Shape: Vertical up and down movement (SF);<br>-Quality: Movement with smooth reversals (SF)	Depressed affect; Positive affect;<br>Vitality
Koch et al.	2016	Embodied Self in Parkinson's disease: How does the experience of beauty influence psychological variables?	34	Quan, one group pre-post trial	Parkinson Outpatient (mostly outpatient)	DMT group session: one-time Tango intervention (2h) by DMT	Experience of beauty (JF); Self-efficacy (CF)	Well-being Body self-efficacy, Expectations (re: treatment method)
Mannheim et al.	2013	Assessing effects of DMT in oncology	118	Quan, one group, pre- post trial	Oncological rehabilitation, female cancer patients	DMT group<br>2-10 weekly sessions of 70 min	The first six factors resulted from factor analysis:<br>-Body/Self-Perception (SF);<br>-Vitality (JF);<br>-Expression of emotion (CF);<br>-Emotional relaxation (JF);<br>-Energy discharge/tension release (SF),<br>-Social Component (CF)	Quality of life; Anxiety; Depression; Self-concept; Well-Being

Author(s)	Year	Study purpose	*N*	Study design	Participants / setting	Intervention characteristics	Therapeutic factors	Outcome(s)
							Getting in touch with the body (SF)<br>Pleasure of movement (JF)<br>Enhancement of self-esteem (CF)<br>Finding inner balance (CF)	
Shim	2015	Identifying factors of DMT that foster resilience in people with chronic pain	16	Qual	Adults with chronic pain, community setting	DMT group chronic pain intervention;<br>10 weekly 70-min group sessions	Kinesthetic awareness (JF);<br>Enactment (SF);<br>Expressivity in movement (SF);<br>Body-mind connection (SF);	Resilience
Shim et al.	2017	Identifying factors of DMT that foster resilience in people with chronic pain	19	Mixed method	Individuals with chronic pain, community health center and osteopathic medicine center	DMT group, 10 weekly sessions of 10 min	Three major factors:<br>1) Loosening up of movement (SF);<br>learning new ways of living in the body (SF)<br>2) Broaden-and-build via experience of positive emotions (CF);<br>-Activating self-agency (JF),<br>-Connecting to self (CF),<br>-Connecting to others (CF),<br>-Enhancing emotional intelligence (CF)<br>-Reframing (CF);<br>3) Therapeutic alliance (CF).	Resilience;<br>Pain intensity;<br>Body awareness;<br>Acceptance;<br>Kinesiophobia;<br>Emotional Health;<br>and others

Author(s)	Year	Study purpose	*N*	Study design	Participants / setting	Intervention characteristics	Therapeutic factors	Outcome(s)
Shim et al.	2019	Testing/developing a theoretical model for therapeutic factors in DMT	20	Mixed method	Individuals with chronic musculoskeletal pain	DMT group chronic pain intervention: 10 weekly 70-min group DMT interventions	Mobilizing/Loosening up in movement (SF); -loosening up on all levels, to make flexible (SF); Broaden-and-built affect (positive emotions support reprocessing and change) (CF); via: -Connecting to self (CF) -Connecting to others (CF); -(Cognitive) Reframing (CF) -Activating self-agency (JF), -Enhancing emotional well-being (CF)	Resilience; Pain intensity;
Shuper-Engelhard et al.	2019	Identify factors that improve relationship quality in couples	28	Qual	Couples therapy	DMT group couples therapy	Mirroring in movement (SF); Creating a safe place (CF); Mentalization (CF)	Body-mind connection
Ventouras et al.	2015	Identifying factors of DMT that help to improve symptoms of schizophrenia	8	Quan, EEG-study (one group, comparing EEG-rhythms)	Patients with schizophrenia	DMT group: primitive expression: 12 PE DMT sessions, 2x per week, for 6 weeks	Intra- and interhemispheric connectivity	-
Wiedenhofer et al.	2017	Investigating the impact of the factor of non-goal-oriented	53	Quan, RCT	Adults with self-reported stress in an academic setting	DMT group: improvisational movement (vs task-related movement); one-	Non-goal orientation of dance movement (SF)	Perceived stress; Body self-efficacy; Well-Being

Author(s)	Year	Study purpose	*N*	Study design	Participants / setting	Intervention characteristics	Therapeutic factors	Outcome(s)
		movement on stress reduction				time intervention, 30 min		
Winther & Stelter	2008	Identifying factors that foster post traumatic growth	1	Qual, phenomenological hermeneutic	Individual therapy of a woman in private practice (trauma)	DMT individual: "Dansergia" (improvisational, energy-releasing intervention); treatment over three years, sessions 1.5h	Facing deeper issue from the past (CF)	Experiential and communicative changes

Note. CF = common factor of psychotherapies, JF = joint factor across CATs disciplines, SF = specific factor in a specific CATs discipline, Qual = qualitative study, Quan = quantitative study, RCT = randomized controlled trial, EEG = The electroencephalogram used to measure electrical activity of the brain.

Table S3

Therapeutic Factors of Drama Therapy (DT) and Psychodrama (PD)

Author(s)	Year	Study purpose	*N*	Study design	Participants / setting	Intervention characteristics	Therapeutic factors	Outcome(s)
Armstrong et al.	2016	Identify dramatic projection and embodiment and their relation to experiencing.	1	Quan<br><br>Process evaluation	Educational setting with one male adult	PD; DT<br>role method; DvT<br>Individual sessions.	Dramatic embodiment (SF);<br>Dramatic projection (SF);<br>Experiencing (CF)	Definitions of experiences
Bucuta et al.	2018	How does PD empower abused women?	33	Mixed methods	Reformed Christian centre for abused women	PD group,<br>25 weekly sessions of 120 min.	Role-reconstruction (SF);<br>Releasing and relief (CF);<br>Understanding the pattern/insight (CF);<br>Hope and optimism (CF);<br>Empowering (CF)	Spontaneity;<br>Psychological distress
Cassidy et al.	2017	How do drama therapists and clients experience therapeutic change processes across DT approaches?	14	Qual	Drama therapists and clients<br><br>Private practices	DT group and individual	Working within a safe distance within or outside the drama (SF);<br>Being allowed and allowing self to play and try out different ways of being (JF);<br>Creating something visible and having physical experiences using the body (JF);<br>Finding a language to communicate (JF);<br>Being actively involved in therapy (JF);<br>Increased reflective functioning (CF)	None
Cassidy et al.	2014	Which change processes are identified across drama therapeutic approaches?	13	Systematic review of 13 case studies	Various settings	DT group and individual	Working in the ‘here and now’ (CF)<br>Establishing safety (CF)<br>Therapist working alongside the client within and outside of the drama (JF)<br>Offering control and choice (JF)<br>Being actively involved (JF)	None

Author(s)	Year	Study purpose	*N*	Study design	Participants / setting	Intervention characteristics	Therapeutic factors	Outcome(s)
Goldstein	1971	Investigating the efficacy of the PD technique doubling.	15	Quan: One-group design. change process only	Hospital- withdrawn psychiatric patients with minimal ability to speech	PD group, 40 twice weekly sessions	Doubling technique (SF)	Increased verbal speech
Kellermann	1987	Assessing which therapeutic factors participants found most helpful.	82	Quan repeated measures within one group	community-based setting	PD group duration not specified	Yalom's therapeutic factors for group therapy (CF), of which most helpful were: Catharsis (SF), Cognitive insight (CF), Interpersonal relationship (CF)	None
Kellermann	1985	Assessing which therapeutic factors participants found most helpful.	30	Survey	Clinical- therapeutic / experiential / didactic groups	PD group, 4 months of sessions	Yalom's therapeutic factors for group therapy (CF), of which most helpful were: self-understanding (CF) Catharsis (CF), interpersonal learning (CF).	None
Kim	2003	Investigating the session-level effect of PD on the protagonist and audience.	12	Quan repeated measures within one group	Counseling center for adolescents	PD, 10 sessions of three hours within 4 days.	Yalom's therapeutic factors for group therapy (CF); Post session evaluation: depth and smoothness (CF); Post session emotional state: disappointment and nervousness (CF).	None
Orkibi et al.	2014	Examining the contribution of PD/DT to the outcomes.	12	Quan repeated measures within one group	Community rehabilitation	DT, PD 20 weekly sessions of 120 min	Surplus reality (SF); Spontaneity (JF); Creativity (JF); Playfulness (JF); Encounter (SF); Role reversal (SF); Witnessing (JF)	Increased Self-esteem; Decreased Self-stigma; Public-stigma
Orkibi et al.	2017a	Examining the association between in-session client measures.	16	Quan One-group design. change process only	Junior high school	PD group, weekly 90 min. psychodrama, 16-22 sessions	Client dramatic engagement (SF); Client involvement (JF); Client in-session behaviors (CF); Client-therapist therapeutic bond (CF)	None
Orkibi et al.	2017b	Investigating the relationship between	40	Quan CCT	Junior high school	PD group, 16-22 weekly	Productive behaviors (CF) Resistance decrease (CF)	Self-concept; Loneliness

Author(s)	Year	Study purpose	*N*	Study design	Participants / setting	Intervention characteristics	Therapeutic factors	Outcome(s)
		process and outcome variables.				sessions of 90 min		
Ozbay et al.	1993	Assessing patients' perception of therapeutic factors.	10	Quan repeated measures within one group	Outpatient psychiatric hospital for youth	PD group, 20 weekly sessions of 120 min	Yalom's therapeutic factors for group therapy (CF)	None
Testoni et al.	2018	Assessing changes in study outcomes and identifying factors that lead to therapeutic change.	4	Mixed method	Therapeutic community for adults with addictions	PD 6 months weekly sessions of 120 min	Self-awareness (CF); Receiving and providing support (CF)	Spontaneity; Psychological distress; Self-efficacy; Hope and optimism.

Note. CF = common factor of psychotherapies, JF = joint factor across CATs disciplines, SF = specific factor in a specific CATs discipline, Qual = qualitative study, Quan = quantitative study, CCT = controlled clinical trial, without randomization.

Table S4

Therapeutic Factors of Music Therapy (MT)

Author(s)	Year	Study purpose	*N*	Study design	Participants / setting	Intervention characteristics	Mechanisms of change	Outcome(s)
Ahonen-Eerikäinen et al.	2007	Exploring the main role of music in MT and identifying the therapeutic factors.	6	Qual	Patients with dementia	MT group, active MT program interventions	Musical engagement (SF) Group cohesiveness (CF); Empowering experiences (CF); Joyful moments (JF);	Dealing with difficult feelings; Personal growth
Ansdell et al.	2010	Eliciting cross-professional interpretations of clinical events from an exemplary single case of a woman with a psychotic illness. Note: N = 5 refers to five raters.	5	Qual Phenomenologic-al study	Outpatient mental health care, patient with psychotic episodes	MT, Nordoff-Robbins method, 2 sessions	Use of tone (SF); Being in the present moment (CF); Musical expansion (SF); Being expressive (JF)	Affect regulation; Psychotic symptoms
Baker et al.	2015	Examining effects of a narrative songwriting program and identifying mechanisms of change	10	Quan repeated measures within one group	Medical setting, patients in recovery from neurological injuries	Narrative songwriting program. 12 sessions of 60 min	Experience of flow (JF); High levels of meaningfulness (CF)	Self-concept; Well-being; Mechanisms of change
Bibb & McFerran	2018	Exploring the role of group singing in regaining healthy relationships with music to promote mental health.	23	Qual, grounded theory	Mental healthcare, community setting, weekly sessions for length of stay	MT group, singing, active MT	Triggering musical encounters (SF)	Coping
Bonde	2005	Exploring metaphors and narrative in one cancer survivor's GMGIM therapy.	1	Qual, Phenomenolo-gical study	Adult female oncology patient, 10 sessions of 10 min	MT group, Bonny Method of Guided Imagery and Music (BMGIM)	Therapeutic alliance (CF); Agency (CF)	Coping skills; Gain insight into life choices; Gain insight into personality characteristics
Carr et al.	2012	Examining effects of MT on PTSD symptoms	17	Quan RCT	Outpatient clinic, adults with PTSD	MT: manualized group improvisation and structured	Structuring/repetitive nature of music (SF);	Distress (in PTSD); Depression (in PTSD)

Author(s)	Year	Study purpose	*N*	Study design	Participants / setting	Intervention characteristics	Mechanisms of change	Outcome(s)
						interventions,10 sessions of 60 min	Point of focus/link to the present (CF);<br>Group cohesiveness (CF)	
Dalton & Krout	2015	Development and implementation of the Grief Song-Writing Process(GSWP) with bereaved adolescents.	123	Qual,<br>Analysis of 123 songs of patients	Mental healthcare, bereaved adolescents	MT group/ind,<br>Songwriting, active / expressive MT,<br>weekly sessions over a longer period	Group cohesiveness (CF);<br>Ownership (CF);<br>Pride (CF)	Facilitating grief;<br>Coping with feelings of grief
De Witte et al.	2020a	Examining overall effects of music interventions on stress-related outcomes	104	SR and two meta-analyses	Medical settings, mental healthcare setting	MT<br>active and receptive interventions	Tempo of the music (60-80bpm) (SF)<br>Group cohesiveness (CF)<br>Distraction of stress-increasing thoughts (JF)	Stress-related outcomes (physiological and psychological)
De Witte et al.	2020b	Examining overall effects of MT on stress-related outcomes	47	SR and meta-analysis	Medical settings, mental healthcare settings	MT,<br>active and receptive interventions	Altering of inherent body-rhythms (SF);<br>Feelings of togetherness and bonding (CF)	Stress-related outcomes (physiological and psychological)
De Witte et al.	2020c	Collecting practice-based knowledge on the most efficient music therapy interventions used by music therapists to reduce stress patients with MID.	13	Qual,<br>focus group study	Mental healthcare institutions for adults with MID	MT group/individual, active MT, improvisation	Musical simplicity (SF);<br>Slow and steady music tempo (SF);<br>Repetitive rhythm (SF);<br>Implementing structure (JF);<br>Safety (CF);<br>Predictability (CF)	Stress release;<br>Relaxation
Gardstrom et al.	2017	Exploring women's perceptions of the usefulness of group MT in addictions recovery.	15	Qual,<br>analysis of 15 group sessions in total	Residential care for women with addiction	MT group,<br>vocal /instrumental re-creation and improvisation,<br>1-5 twice weekly sessions of 50 min	Intramusical connections (SF);<br>Group cohesiveness (CF);<br>Strengthen sense of self (CF);<br>Enjoyment (JF);<br>Catharsis (CF);<br>Altruism (CF);<br>Instillation of hope (CF);<br>Interpersonal learning (CF);	Mood;<br>Affect

Author(s)	Year	Study purpose	*N*	Study design	Participants / setting	Intervention characteristics	Mechanisms of change	Outcome(s)
							Impacts present energy level (CF)	
Goldberg et al.	1988	Evaluating similarities and differences in therapeutic processes between MT and verbal group therapy.	61	Quan CCT	University based hospital, short term treatment unit for psychiatric patients	MT group improvisation, music listening. 5 weekly sessions of 50 min	Feelings of cohesiveness (CF); Interpersonal learning (CF) through the structuring nature of music (SF);	Curative factors (what was helpful; Patient satisfaction; General mental health
Kellet et al.	2019	Testing whether G-CAMT could improve relational and empathic abilities and reduce dissociative symptoms	20	Quan quasi-experimental	Mentally disordered male offenders, high secure hospital	Group cognitive analytic music therapy (G-CAMT), Improvisation-based, 16 sessions of 90 min	Creative self-expression (JF); Musical dialogue (SF); Verbal dialogue (CF)	Relational dynamics
Landis-Shack et al.	2017	Exploring music therapy for posttraumatic stress in adults	4	Theoretical review (4 empirical studies included)	Adult PTSD patients,	MT group, active and receptive interventions	Engagement with the musical experience (SF); Physical act of music making (SF); Musical cues that are used to ground and modulate distress (SF); Group cohesiveness (CF)	Reduced impact of distressing reminders; Mood regulation; Modulate distress
McDermott et al.	2013	Exploring effects and applicability of MT for people with dementia	18	Systematic narrative review (18 studies included)	Studies included with patients with moderate to severe dementia	MT improvisation, receptive relaxation, small groups	Music stimulated autobiographical recall (SF); Musical attunement (SF); Non-verbal attunement (JF); Reminiscence (CF); Meaning (CF); Physiological changes	Depressive symptoms; Anxiety; Behavioural and psychological symptoms; Short-term agitation
Millet & Gooding	2017	Examining whether MT is effective in reducing preoperative anxiety in pediatric patients	40	Quan RCT	Medical pediatric ambulatory surgery unit	Distraction-based MT based on musical-alternate engagement and music-assisted relaxation.	Music as motivating (CF); Structuring nature of music (SF); Active engagement (JF)	State-anxiety

Author(s)	Year	Study purpose	*N*	Study design	Participants / setting	Intervention characteristics	Mechanisms of change	Outcome(s)
Passiali	2012	Investigating mutually responsive orientation behaviors of young children and their family members during MT.	4	Qual, grounded theory, including 4 families	Families with low income and history of maternal depression	Family-based MT, song games, songwriting, music assisted relaxation	Playfulness involved in playing/sharing instruments (SF); Musical interactions (SF); Joint attention (CF); Modelling (CF); Turn taking (CF)	Harmonious parent-child communication; Encouraging intimacy and connection
Porter et al.	2017	Examining the processes and experiences involved in the introduction of MT as a complementary therapy to palliative care.	16	Qual, Realist evaluation study	Palliative care	MT individual, active / receptive MT interventions, twice weekly sessions of 60 min for 3 weeks.	Playfulness by playing instruments (SF); Shared musical experiences (SF); Reframing identity (CF); Temporary distraction from illness (JF); Reconnect with key moments (CF)	Emotional well-being; Mood; pain; Anxiety
Potvin et al.	2018	Enhancing understanding of informal hospital caregivers' needs during pre-bereavement and examining the role of MT sessions for caregivers and recipients.	14	Qual, Grounded theory	Palliative care	MT group, resource-oriented active MT	Artistic agency (JF); Musical synchronicity (SF);	Balanced caregiver's role
Robb	2000	Exploring effects of MT on behaviour of hospitalized children in isolation and developing a contextual support model.	10	Quan within-subjects design (ABCA)	Paediatric hospital unit for paediatric oncology patients	MT individual with family involvement, Instrument playing, choice making, 1 session	Offering music choices (SF) Structuring /safe nature of music (SF); Supportive /familiar atmosphere (CF); Agency (CF)	Engaging; Behavioural responses; Autonomy support; Self-regulation
Rolvsjord	2010	Exploring what patients do to make MT work.	7	Qual Multiple case study	Inpatient psychiatry	MT, improvisation, active and expressive MT	Agency in music making (SF); Creativity (JF); Personal value (CF)	Affective change in severe mental health symptoms
Short et al.	2009	Exploring how cardiac patients respond to BMGIM sessions and how the music may	6	Qual	Medical setting,	MT group, receptive MT,	Programmed classical music (SF); Deep relaxation (JF);	Emotional responses; Physical responses

Author(s)	Year	Study purpose	*N*	Study design	Participants / setting	Intervention characteristics	Mechanisms of change	Outcome(s)
		contribute to emotional and physical meanings during rehabilitation.			adult patients recovering from cardiac surgery	Bonny Method of Guided Imagery and Music (BMGIM). 31 sessions, 6 per week.	Interpreted symbols /images (JF)	
Waldon	2001	Examining effects of MT on mood states and cohesiveness in adult oncology patients	10	Quan<br>CCT comparison of 2 MT conditions	medical setting, cancer diagnosed patients	MT group,<br>music making and music responding.<br>10 session of 60 min	Group cohesion (CF);<br>Validating feedback of group members (CF)	Mood
Warth et al.	2016	Examining cardiovascular responses to receptive music therapy in palliative care	84	Quan<br>RCT	Adult Palliative care unit,	MT group,<br>Receptive MT,	Verbal and non-verbal skills (CF);<br>Therapeutic relationships (CF);<br>Prosocial behaviour (CF)	Modulation of cardiac activity

Note. CF = common factor of psychotherapies, JF = joint factor across CATs disciplines, SF = specific factor in a specific CATs discipline, Qual = qualitative study, Quan = quantitative study, RCT= randomized controlled trial, CCT = controlled clinical trial, without randomization.

Table S5

Therapeutic Factors in Studies with More than One CATs Modality

Author(s)	Year	Study purpose	*N*	Study design	Participants / setting	Intervention characteristics	Change factor / mechanisms of change	Outcome(s)
Chiang et al.	2019	Examining if CATs decrease mental health symptoms.	86	Narrative Review<br><br>(86 studies included)	SMI (= severe mental illness, i.e., trauma, schizophrenia, major depression, and bipolar disorder) in diverse mental health settings,<br>86 studies	CATs (AT, MT, DMT, DT/PD , expressive writing)	Use of non-verbal medium for expression (JF);<br><br>Creativity (JF);<br><br>Developing artistic talents (JF);<br><br>Comfortable & liberating<br><br>environment (CF);<br><br>Offering therapeutic activity (JF);<br><br>Levels of trust in healthcare providers and treatment plans (CF);<br><br>Molecular biological factors*	Self-esteem; Coping; Mood; Cognitive functioning; Social functioning;<br>Reduction of SMI symptoms
Dunphy et al.	2019	Examining which outcomes, processes, and mechanisms of CATs address depression in older adults.	75	Systematic Review<br><br>(75 studies included)	Older adults with depression, diverse group settings, interventions most effective when conducted by a CAT	AT, DMT, DT and MT interventions of typically one hour were held 1-2x a week over periods of 12 - 52 weeks. typically as group format, with	Intra-personal<br>Enhanced self-concept (CF);<br>Strengthened agency and mastery (CF);<br>Processing/communication of emotions (CF)	Depression;<br>Reduced symptoms

Author(s)	Year	Study purpose	*N*	Study design	Participants / setting	Intervention characteristics	Change factor / mechanisms of change	Outcome(s)
						specific attention to individual group members	Cultural creative expression (JF);<br>Aesthetic pleasure (JF)<br>Cognitive stimulation of memory (CF)<br>Social increased social skills and connection (CF)<br>Physical increased muscle strength; neurochemical effects, eg endorphin release	
Heynen et al.	2019	Examining if CATs reduce symptoms of depression and anxiety via the therapeutic alliance.	167	Quan Repeated measures, one group design	Clinical inpatient setting; mental health diagnoses (e.g., anxiety disorders, depression, substance abuse, personality disorder	AT, MT, Psychomotor Therapy by a qualified CAT therapist	Therapeutic alliance (CF)	Depression; Anxiety
Kalaf & Plante	2019	Exploring if expressive art therapy enhances resilience.	10	Qual (interviews, observation, parental or facilitator feedback)	Syrian war refugees, 12-16 years old, elementary school	Expressive art therapy workshop (10 days); goal: collaborative production of an animated movie on resilience provided by art therapist	Experiencing positive affect (JF);<br>Developing supportive relationship (CF);<br>Community engagement (CF);<br>Empowerment (CF);<br>Meaning making (CF);	Resilience
Parsons et al.	2020	Exploring client-reported helpful factors within arts psychotherapies.	17	Qual systematic review using thematic synthesis, pluralistic meta-approach	Adults with depression	arts psychotherapies (art, music, drama, and dance movement psychotherapy as well as NICE recommended	Working with the here and now (CF);<br>Getting to the root (CF);<br>Tailored structure and content to fit the client (CF);<br>Client targets (CF);	Depression

Author(s)	Year	Study purpose	*N*	Study design	Participants / setting	Intervention characteristics	Change factor / mechanisms of change	Outcome(s)
				(17 arts therapies studies included)		forms of psychotherapy, i.e., CBT, Counselling for Depression and Short-term Psychodynamic Psychotherapy)	Fundamental relational skills and features (CF); Encouraging active engagement (JF); Bringing it all together (CF)	
Schiltz	2014	Does multimodal arts psychotherapy (literary and musical production) affect mentalization, reduction of anxiety, and negative feelings? How does it contribute to the diagnostic process and the epistemological knowledge on CATs workings?	56	Quan	Adolescents suffering from conduct disorders, regular school setting	Music therapy and expressive writing (Multimodal Arts Psychotherapy):<br>- free musical and vocal improvisation,<br>- stories written under musical induction<br>- verbal elaboration	Enhancement of imaging and symbolic elaboration through stories written under musical induction (JF);<br>Release of intrapsychic tension through musical improvisation (SF);<br>Enhancement of control and concentration through musical improvisation (SF);<br>Enhancement of self-knowledge through verbal elaboration of artistic product (JF)	Mentalization; Anxiety; Regulation of negative feelings; reducing acting out; Concentration
Sjöström-Flanagan	2004	How can metaphors emerging from DMT and AT (be the link to) support the body-mind integration and coping with chronic pain?	153	Qual (patient interviews based on 500+ metaphors that emerged from movement and art within 3 years)	clients with chronic pain who already went through physiotherapy and verbal therapy (fibromyalgia and other)	3-year project Form and Freedom (DMT+ AT); Part 1 (n=110): 9-week program, one 2h session per week using music, art, imagery, relaxation;<br>Part 2 (n=61): ongoing group,	Metaphor (JF);<br>Awareness of ego function/ self-realization (CF);<br>Personal responsibility (CF);<br>Presence in the moment (CF)	Acceptance of chronic pain;<br>Quality of life

Author(s)	Year	Study purpose	*N*	Study design	Participants / setting	Intervention characteristics	Change factor / mechanisms of change	Outcome(s)
						1x2h per week, up to 1.5 years; freer exploration of own movement process		

Note. CF = common factor of psychotherapies, JF = joint factor across CATs disciplines, SF = specific factor in a specific CATs discipline, Qual = qualitative study, Quan = quantitative study, RCT= randomized controlled trial, CCT = controlled clinical trial, without randomization. NICE = National Institute for Health and Care Excellence in England, CBT = cognitive behavioral therapy.

CATs= creative arts therapies, AT= art therapy, DMT = dance movement therapy, DT= drama therapy, MT = music therapy, PD= psychodrama.
